# Hybrid external-cavity lasers (ECL) using photonic wire bonds as coupling elements

**DOI:** 10.1038/s41598-021-95981-w

**Published:** 2021-08-12

**Authors:** Yilin Xu, Pascal Maier, Matthias Blaicher, Philipp-Immanuel Dietrich, Pablo Marin-Palomo, Wladislaw Hartmann, Yiyang Bao, Huanfa Peng, Muhammad Rodlin Billah, Stefan Singer, Ute Troppenz, Martin Moehrle, Sebastian Randel, Wolfgang Freude, Christian Koos

**Affiliations:** 1 Institute of Photonics and Quantum Electronics (IPQ), Karlsruhe Institute of Technology (KIT), Engesserstrasse 5, 76131 Karlsruhe, Germany; 2 Institute of Microstructure Technology (IMT), KIT, Hermann-von-Helmholtz-Platz 1, 76344 Eggenstein-Leopoldshafen, Germany; 3 Vanguard Automation GmbH, Gablonzer Strasse 10, 76185 Karlsruhe, Germany; 4 Fraunhofer Institute for Telecommunications, Heinrich Hertz Institute (HHI), Einsteinufer 37, 10587 Berlin, Germany

**Keywords:** Integrated optics, Optoelectronic devices and components, Semiconductor lasers, Lithography

## Abstract

Combining semiconductor optical amplifiers (SOA) on direct-bandgap III–V substrates with low-loss silicon or silicon-nitride photonic integrated circuits (PIC) has been key to chip-scale external-cavity lasers (ECL) that offer wideband tunability along with small optical linewidths. However, fabrication of such devices still relies on technologically demanding monolithic integration of heterogeneous material systems or requires costly high-precision package-level assembly, often based on active alignment, to achieve low-loss coupling between the SOA and the external feedback circuits. In this paper, we demonstrate a novel class of hybrid ECL that overcome these limitations by exploiting 3D-printed photonic wire bonds as intra-cavity coupling elements. Photonic wire bonds can be written in-situ in a fully automated process with shapes adapted to the mode-field sizes and the positions of the chips at both ends, thereby providing low-loss coupling even in presence of limited placement accuracy. In a proof-of-concept experiment, we use an InP-based reflective SOA (RSOA) along with a silicon photonic external feedback circuit and demonstrate a single-mode tuning range from 1515 to 1565 nm along with side mode suppression ratios above 40 dB and intrinsic linewidths down to 105 kHz. Our approach combines the scalability advantages of monolithic integration with the performance and flexibility of hybrid multi-chip assemblies and may thus open a path towards integrated ECL on a wide variety of integration platforms.

## Introduction

﻿Tunable semiconductor lasers are key building blocks of integrated optics. Among the various approaches, external-cavity lasers (ECL) are particularly promising, combining direct-bandgap III–V materials that offer broadband optical gain with passive external feedback circuits that may be tuned over a wide wavelength range. The feedback circuits may be realized on advanced photonic integration platforms such as silicon photonics (SiP)^1–8^ or silicon nitride^9–12^, thereby offering a direct route towards efficient co-integration of the ECL with other highly functional building blocks. Recent demonstrations of integrated ECL rely on two main approaches: heterogeneous integration^2–4,12,13^, where dies of III–V gain materials are bonded onto passive waveguides for further front-end of line (FEOL) processing on a wafer scale, or hybrid integration, where readily processed III–V semiconductor optical amplifiers (SOA) are attached to passive feedback circuits in a back-end of line (BEOL) assembly process^5–11,14^. While heterogeneous integration paves a path towards highly scalable production using, e.g., advanced micro-transfer printing processes^13,15–17^, the associated technical complexity is still considerable. In particular, ultra-clean and extremely smooth surfaces are required, along with precise control over materials and environmental conditions^18^. Moreover, heterogeneous integration makes it difficult to test individual components prior to integration into more complex systems and hence requires tight process control to maintain high yield. Consequently, this approach is mainly suited for high-volume applications that justify the associated technological overhead. In addition, heterogeneously integrated optical gain material may consume considerable space on the SiP chip, and heat sinking is challenging due to the high thermal resistance of the III–V-to-Si bonding layer and the buried oxide^19^. Hybrid integration, in contrast, can overcome these limitations, is non-invasive to the front-end fabrication process flow of the passive external-cavity circuit^20^, and can thus be applied to a wide range of integration platforms such as SiP^5–8^, silicon nitride^9–11^, or planar lightwave circuits (PLC)^7,14^. However, the concept crucially relies on high-precision assembly with tolerances in the lower micrometer or even sub-micrometer range, often requiring slow and expensive^21^ active alignment techniques to achieve low-loss coupling. Scalability to high production volumes is therefore limited.

In this paper, we demonstrate a new class of hybrid external-cavity lasers (ECL) that do not require any high-precision alignment techniques. Instead, the devices rely on 3D-printed polymer waveguides, so-called photonic wire bonds (PWB)^22–24^, that connect the active III–V gain die to an external feedback circuit on a SiP chip. In this approach, the shape of the PWB can be adapted to the actual positions of the chip facets at both ends, thereby compensating for placement inaccuracies. In a proof-of-concept experiment, we demonstrate a hybrid ECL with a tuning range of more than 50 nm, a side mode suppression ratio (SMSR) above 40 dB, and an intrinsic linewidth of 105 kHz. The process of photonic wire bonding can be efficiently automated and allows to connect photonic dies with vastly different mode-field sizes, thereby making the concept compatible with a wide range of integration platforms. We believe that our approach has the potential to offer a route towards advanced ECL that combine the flexibility of hybrid integration with the scalability of waver-level heterogeneous integration concepts.

## Results and discussion

### Device concept

**Figure 1:**
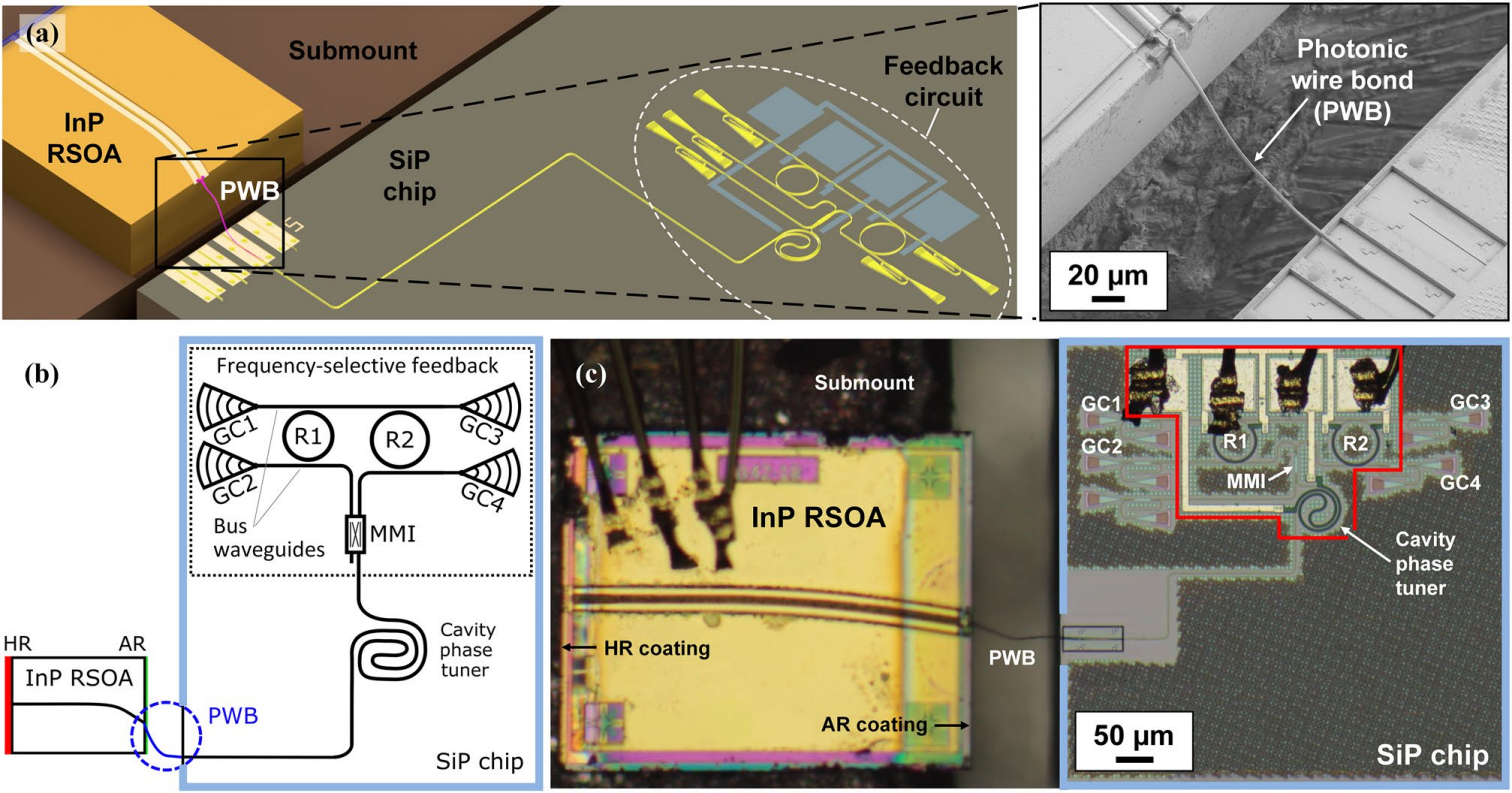
Concept and realization of a hybrid ECL with a photonic wirebond (PWB) as intra-cavity coupling element. (**a**) Concept: The device consists of an InP-based reflective semiconductor optical amplifier (RSOA) that is connected to a thermally tunable feedback circuit on a silicon photonic (SiP) chip. A photonic wire bond (PWB, see Inset) connects the facet of the RSOA to the SiP external-cavity feedback circuit. The assembly is built on a metal submount that simultaneously acts as an efficient heat sink. (**b**) Building blocks of the device realized for our proof-of-concept demonstration. The 600 µm-long RSOA is equipped with a high-reflectivity (HR) coating at the back facet. The front facet is angled (tilt angle 9°) and anti-reflection-(AR)-coated with respect to polymer (refractive index *n* = 1.56). The SiP external-cavity circuit (framed in light blue) comprises a 350 µm-long spiral-shaped phase shifter for adjusting the intra-cavity phase (cavity phase tuner) and a tunable frequency-selective feedback structure. Frequency selectivity is provided by two symmetrically coupled Vernier ring resonators R1 and R2 in add-drop configuration with diameters *D*_1_ = 62 µm (R1) and *D*_2_ = 67 µm (R2) and with coupling gaps of 180 nm. Note that the cascaded Vernier rings can be assumed to be reciprocal, such that the feedback circuit behaves similarly to a Sagnac loop mirror. The lower left-hand input port of the 2 × 2 MMI can thus in principle be left unconnected, and the associated grating coupler shown in Subfigure (**a**) was omitted here for simplicity. (**c**) Microscope image of the assembled device. Left-hand side: InP RSOA; right-hand side: SiP chip (framed light blue). The relevant part of the external-cavity circuit is marked by a red bounding box.

The concept of a hybrid ECL with a photonic wire bond (PWB) as chip-to-chip coupling element is shown in Fig. 1. The device consists of an InP-based reflective semiconductor optical amplifier (RSOA) that is coupled to a thermally tunable feedback circuit on a silicon photonic (SiP) chip, see Fig. 1a. The 3D-printed PWB shown in the Inset of Fig. 1a allows to connect the edge-emitting RSOA to an adiabatic down-tapered strip waveguide^22^ on the surface of the SiP chip—without the need for any high-precision assembly techniques. The RSOA can be directly mounted onto a metal heat sink, thereby ensuring efficient cooling of the device. A more detailed description of the assembly built in the course of our experiments is given in Fig. 1b. The RSOA is 600 µm long and has a high-reflectivity (HR) coating at the back facet, while the front facet is angled and coated with an anti-reflection (AR) layer. The external-cavity circuit on the SiP chip consists of a 2.2 mm-long strip waveguide (WG), that includes a 350 µm-long thermally tunable spiral-shaped phase-shifter for adjusting the cavity phase, and of a tunable frequency-selective feedback structure. The feedback structure relies on a Vernier pair of thermally tunable ring resonators R1 and R2 in add-drop configuration, where each ring is coupled to two bus waveguides. A multi-mode interference (MMI) coupler is used to split and combine the signals propagating through the feedback structure. The silicon chip was fabricated in a standard silicon photonics process using 248 nm deep-UV lithography. Figure 1c shows a top view of the fabricated ECL assembly. Compared to the InP RSOA, the relevant part of the external-cavity circuit, marked by a red bounding box, is rather small.

### Component characterization

To fully evaluate our integration concept, all components of the ECL are individually characterized prior to assembly. The ability to start the assembly from fully characterized known-good components highlights one of the key advantages of our hybrid approach as compared to heterogeneous integration concepts. The following sections describe the measured performance of the RSOA and of the feedback circuit.

#### RSOA

**Figure 2:**
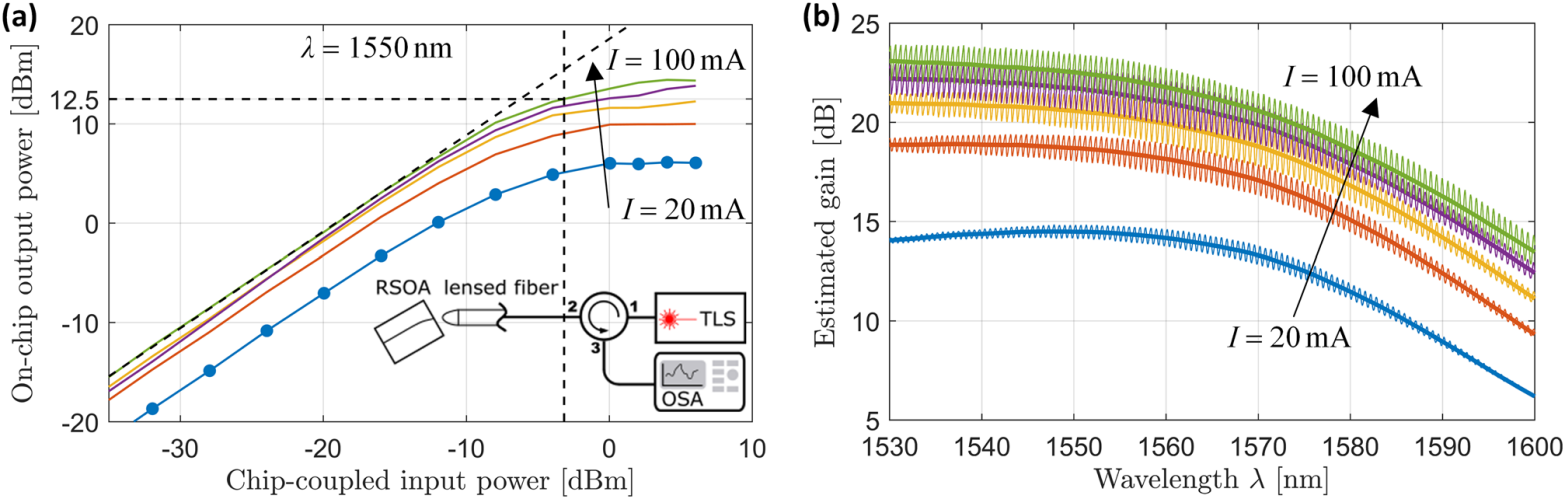
RSOA characterization. (**a**) Typical on-chip output power vs. chip-coupled input power for various injection currents *I*. In the experiment, the device was tested with 12 discrete input power levels—for the sake of readability, the corresponding measurement points are indicated for the curve *I* = 20 mA only. At a bias current of 100 mA, the saturation output power, defined by a 3 dB gain compression, amounts to 12.5 dBm. Inset: Simplified sketch of the underlying experimental setup, consisting of a tunable laser source (TLS), an optical circulator, and an optical spectrum analyzer (OSA). Details of the data evaluation can be found in Supplementary Section 1. (**b**) Typical RSOA gain as a function of wavelength for various injection currents *I*. The ripples stem from reflections at the facets of the 600 µm-long RSOA waveguide and have a periodicity of 73 GHz. Note that the ripple height does not only depend on the gain, but also exhibits a wavelength dependence. We attribute this to the residual reflectivity of the anti-reflection (AR) coating on the front facet of the RSOA, which was designed for coupling to polymer with a refractive index of 1.56 and which leads to stronger residual reflections when operating the device in air as in this experiment. The residual reflectivity of the AR coating reaches a minimum at around 1530 nm, leading to an effective suppression of ripples at this wavelength, despite the comparatively high gain. Towards bigger wavelengths, the ripples naturally reduce with gain. At a bias current of 100 mA, the near-maximum small-signal gain at $\lambda = 1550\;{\text{nm}}$ amounts to 23 dB. Note that the measurement data shown in Subfigure (**a**) and (**b**) was taken from two distinct devices with nominally identical parameters, fabricated on the same wafer.

The 600 µm long C-band RSOA has a back facet with a HR coating (90% reflectivity with respect to air) and an angled front facet ($9^\circ$) with AR coating designed for emission into polymer (*n* = 1.56). The typical measured input/output power characteristics and gain spectra for different currents are shown in Fig. 2. All power levels refer to the on-chip power. At a bias current of 100 mA, the saturation output power, defined by a gain compression of 3 dB, is 12.5 dBm, Fig. 2a, while the near-maximum small-signal gain at $\lambda = 1550\;{\text{nm}}$ is 23 dB, Fig. 2b. All measurements were taken using a lensed fiber. Separate reference measurements were performed to correct for the coupling loss of the lensed fiber, see Supplementary Section 1 for details on the RSOA characterization. Note that the data shown in Fig. 2a,b was taken from two distinct devices with nominally identical parameters, fabricated on the same wafer.

#### Feedback circuit

Frequency selective optical feedback is provided by two symmetrically coupled Vernier ring resonators R1 and R2 in add-drop configuration with diameters 62 µm (R1) and 67 µm (R2), see Fig. 1b. For stand-alone ring resonators, asymmetric coupling can be used to achieve critical coupling when sending light through one of the bus waveguides. In contrast to this, our devices rely on symmetrical coupling, because light is sent into the ring simultaneously from the top and bottom waveguide. Both rings have equal waveguide cross-sections, therefore identical propagation constants, and slightly different free spectral ranges (FSR). The individual ring resonators are characterized by through-port power-transmission measurements via grating couplers GC 1 and GC 3, see Fig. 1b and Supplementary Section 2. The measured data are then fit by a model according to Supplementary Eq. (S2). At a wavelength of 1550 nm, we extract FSR of 368.2 GHz and 340.7 GHz for R1 and R2, respectively, along with approximately equal Q-factors of roughly 28,000 for the coupled resonators. The complete set of fitted parameters is given in Supplementary Section 2.

**Figure 3:**
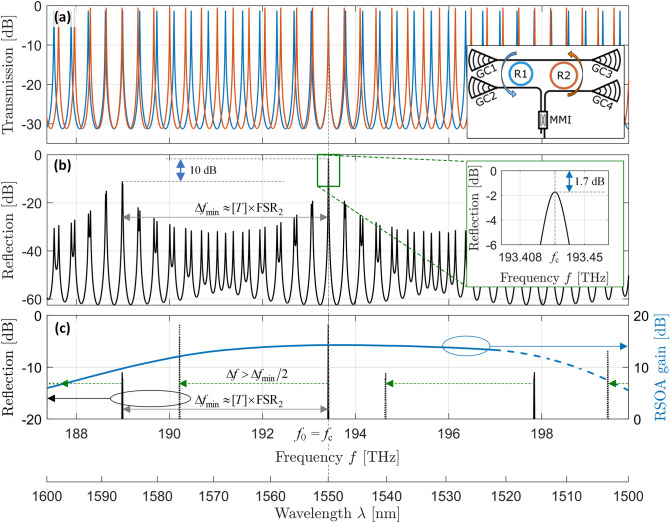
Frequency-dependent reflection characteristics of the external-cavity circuit calculated with the parameters extracted from through-measurement (GC 1 → GC 3), see Supplementary Section 2. (**a**) Drop-port transmission of the individual rings. (**b**) Overall reflection spectrum of the external-cavity circuit. The main peak height indicates a minimum on-chip reflection loss of 1.7 dB, see Inset. (**c**) Exemplary gain spectrum of the RSOA according to Fig. 2b for an injection current of *I* = 20 mA (blue line) with schematic resonance peaks (black lines) as in (**b**). The blue solid line corresponds to measured values of the RSOA gain, whereas the blue dashed line is an estimated extrapolation towards higher frequencies. The solid black lines indicate the strong main reflection peak at the target frequency *f*_0_, which, for the depicted case, corresponds to the center frequency (*f*_c_ = 193.2 THz; *λ*_c_ = 1550 nm) of the gain spectrum, along with the most prominent side peaks that are most prone to unwanted lasing. The dotted black lines indicate the corresponding peaks after a detuning by $\Delta f > \Delta f_{\min } /2$ where $\Delta f_{\min } \approx [T] \times {\text{FSR}}_{2}$, indicated by green arrows.

The two rings of slightly different FSR form a Vernier pair such that the external-cavity circuit offers significant optical feedback only if two ring resonances coincide sufficiently well. To quantify the frequency-selective feedback, we calculate the drop-port transmission of each individual ring, indicated in blue and orange in Fig. 3a and in the associated Inset. To this end, we use Supplementary Eq. (S5) along with the fit values from the previous through-port measurements for each individual ring. For the plot in Fig. 3a, we assume that the rings are tuned to maximum transmission at the center wavelength of the gain spectrum, $\lambda_{{\text{c}}} = 1550\;{\text{nm}}$, such that the calculated transmission resonances coincide at this wavelength. The overall frequency-selective reflection results from multiplication of the individual drop-port power transmission spectra and is shown in Fig. 3b. The peak height at the common resonance indicates the loss which occurs during propagation through the pair of ring resonators. In our case, we find a value of 1.7 dB.

A key parameter of an ECL is the achievable frequency tuning range $\Delta f_{{{\text{tun}}}}$ for emission into a single longitudinal mode. For feedback circuits based on Vernier rings, a large $\Delta f_{{{\text{tun}}}}$ can be achieved without the need for exceedingly small ring resonators, which would lead to increased bending loss and smaller Q-factors. Nevertheless, $\Delta f_{{{\text{tun}}}}$ is usually limited by the finite Q factor of the rings and the associated non-zero resonance widths, which can lead to significant optical feedback even for an imperfect overlap of two closely spaced resonance peaks. In combination with a spectrally non-uniform and possibly inhomogeneously broadened gain spectrum, this feedback could allow lasing at an unwanted secondary longitudinal mode. We estimate a lower bound $\Delta f_{\min }$ for the achievable tuning range $\Delta f_{{{\text{tun}}}}$ by calculating the spectral spacing between the strong main reflection peak and the two most prominent side peaks that are most prone to generate unwanted lasing modes, see Fig. 3b. To this end, we use the so-called tuning enhancement factor *T*, which quantifies the increased tuning efficiency of the Vernier rings in comparison to a single ring resonator. For the Vernier pair, the difference in FSR is by far smaller than the FSR of each individual ring. Hence, only a small refractive-index tuning is required to line up adjacent peaks. Compared to the refractive-index change required to tune a single ring across one FSR, this corresponds to a tuning enhancement factor^25,26^ of

1 $$T = \frac{{{\text{FSR}}_{1} }}{{\Delta {\text{FSR}}}},\quad \quad \Delta {\text{FSR}} = {\text{FSR}}_{1} - {\text{FSR}}_{2} > 0$$

The tuning enhancement factor *T* is at the same time closely related to the frequency spacing between the main reflection peak and the most prominent side peaks that arise from nearly-overlapping resonances of the individual rings. Assuming that the main peak consists of two perfectly aligned resonances at the target frequency $f_{{0}}$, the next side peaks would appear at $f_{0} \pm \Delta f_{\min }$, $\Delta f_{\min } \approx [T] \times {\text{FSR}}_{2} \approx [T - 1] \times {\text{FSR}}_{1}$, where [.] denotes a rounding operation to the nearest integer, see Fig. 3c and Supplementary Section 3 for details. To simplify the estimation of the achievable tuning range, we assume a homogeneous gain spectrum in the following that is broad enough and thereby does not directly limit $\Delta f_{{{\text{tun}}}}$. If the target frequency $f_{0}$ is identical or close to the center frequency $f_{{\text{c}}}$ of the gain spectrum, lasing will occur at $f_{0}$ only, because the side peaks experience less gain as well as higher reflection loss, see Fig. 3c. If the target frequency is tuned away from the center of the gain spectrum, lasing may also occur at the side peaks. Assuming that the gain spectrum is approximately symmetric with respect to its center frequency $f_{{\text{c}}}$ and that the side peaks have the same height as the main peak, a shift by $\pm \Delta f_{\min } /2$ would position two resonance peaks of equal height symmetrically to the center frequency of the gain spectrum and would hence lead to lasing in the first side peak. Under these simplifying assumptions, the minimum tuning range would be slightly less than ${{ \pm \Delta f_{{{\text{min}}}} } \mathord{\left/ {\vphantom {{ \pm \Delta f_{{{\text{min}}}} } 2}} \right. \kern-\nulldelimiterspace} 2}$.

In our device, the side peaks of the reflection spectrum at $f_{0} \pm \Delta f_{\min } /2$ are about 10 dB lower than the main peak. In comparison to this, the gain roll-off at $f_{{\text{c}}} \pm \Delta f_{\min } /2$ is around 3 dB only. We hence find a bigger tuning range, $\Delta f_{{{\text{tun}}}} > \Delta f_{\min }$. This is illustrated in Fig. 3c, where the dotted lines indicate a reflection spectrum which is shifted by $\Delta f > \Delta f_{\min } /2$. We confirm this aspect experimentally: From the passive characterization of our device, we find $T = 13.4$ and $\Delta f_{\min } \approx 4.4\;{\text{THz}}$, corresponding to $\Delta \lambda_{\min } \approx 35\;{\text{nm}}$ at a wavelength of 1.55 µm, while our experiments exhibit a single longitudinal lasing mode over a tuning range between 191.7 THz (1565 nm) and 198.0 THz (1515 nm), i.e., over $\Delta f_{{{\text{tun}}}} = 6.33\;{\text{THz}}$ ($\Delta \lambda_{{{\text{tun}}}} = 50\;{\text{nm}}$), see section “Functional demonstration and characterization” below.

### Module assembly

**Figure 4:**
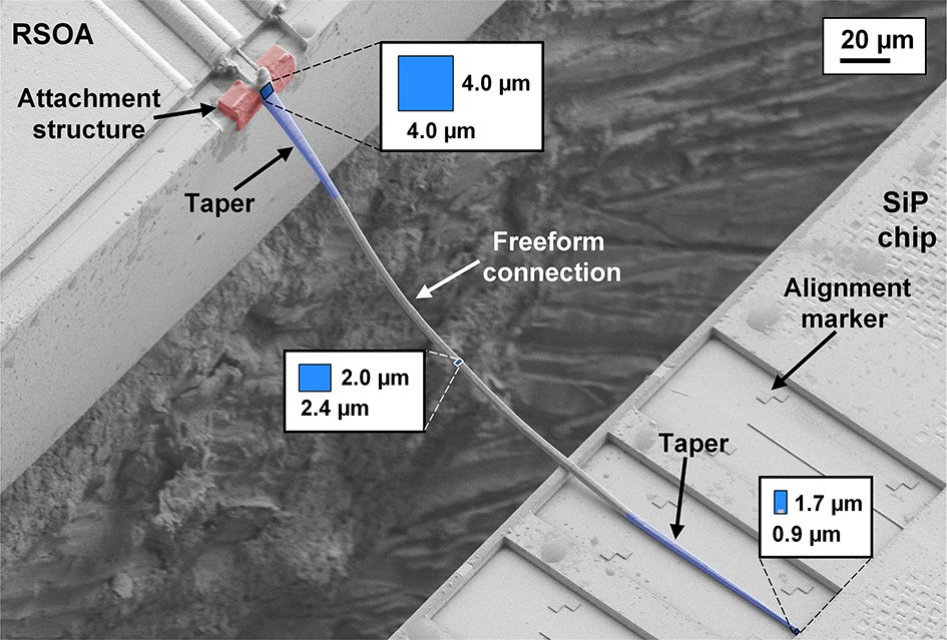
False-colored SEM picture of the fabricated PWB between the InP RSOA and the SiP external feedback circuit. The fabricated cross-section in each part of the PWB is indicated. On the InP side, a taper (blue) is used to transform the mode field on the RSOA facet to that of the freeform PWB connection. At the SiP chip, a polymer-to-silicon double-taper transition^22,23^ is used for efficient connections. The blue rectangles indicate the cross sections at the respective positions along the PWB trajectory. An additional attachment structure (red) is added on the RSOA facet for mechanical stability. Alignment markers on the SiP chip facilitate exact localization of the coupling interface.

For assembly, the RSOA is glued to a copper heat sink using thermally conductive silver-filled glue (EPO-TEK H20E). The copper heat sink and the silicon chip are then coarsely aligned to each other and glued to a common metal submount. The submount contains a step that has been designed such that the chip surface on the RSOA side is approximately 60–80 µm above the chip surface on the Si side, which turned out to allow for a convenient 3D routing of the PWB. An SEM picture of the fabricated PWB is shown in Fig. 4. The PWB trajectory accounts for the chip positions and the oblique emission from the angled RSOA facet. At the interface to the InP RSOA, the PWB comprises a taper with an initial cross-section of 4 µm × 4 µm, matched to the mode-field diameter on the InP side. This cross section is then linearly tapered to that of the freeform section of the bond (2.4 µm × 2.0 µm). At the SiP chip, a polymer-to-silicon double-taper transition^22,23^ connects the PWB to a standard SiP strip waveguide with 500 nm width and 220 nm height. This double taper contains a down-tapered silicon waveguide with a tip width of 180 nm. An additional attachment structure, depicted in red, is added on the RSOA facet for mechanical stability.

Figure 1c shows a microscope image of the fully assembled ECL module. Note that the refractive index of the cured resist ($n_{{\text{r}}} = 1.53$) does not perfectly match the refractive index for which the AR-coating of the RSOA is designed ($n = 1.56$), but the influence is negligible. We estimate a PWB loss of $(2.1 \pm 0.2)\;{\text{dB}}$ from amplified spontaneous emission (ASE) measurements before and after module assembly, see Supplementary Section 1 for details. The estimated PWB loss is slightly higher than previously published results^22,23^, but is still on par with many results demonstrated with butt coupling and active alignment^7,8,27^. We attribute the additional loss mainly to a non-optimum design of the inverse taper on the SiP chip and to the fact that the waveguide was operated in air rather than in a low-index cladding, which would render the curved freeform connection single mode and thus decrease the insertion loss. Note also that the design of the taper towards the facet of the InP chip is based on a mode-field measurement of the RSOA, which was performed using an infrared microscope with an air objective. In contrast to this, the device is finally operated with the facet in direct contact to the polymerized resist of the PWB, having a refractive index of $n_{{\text{r}}} = 1.53$*.* Since the mode field is of the order of the vacuum wavelength, the emission from the RSOA facet might depend on the refractive index of the adjacent medium, and the microscope images captured in air might lead to an over-estimation of the mode-field diameter that is effective when the facet is in contact with resist. This effect might be taken into account in the next device generation, e.g., by performing the mode-field measurement under immersion. Another source of additional loss is the fact that the relative position of the reference points on the surface of the InP chip and the emission spot at the facet might be subject to tolerances due to finite overlay accuracy of the lithography layers used during chip fabrication. For a batch of RSOA from the same chip, which should all be subject to the same offset, this effect might be measured and taken into account during PWB fabrication.

### Functional demonstration and characterization

**Figure 5:**
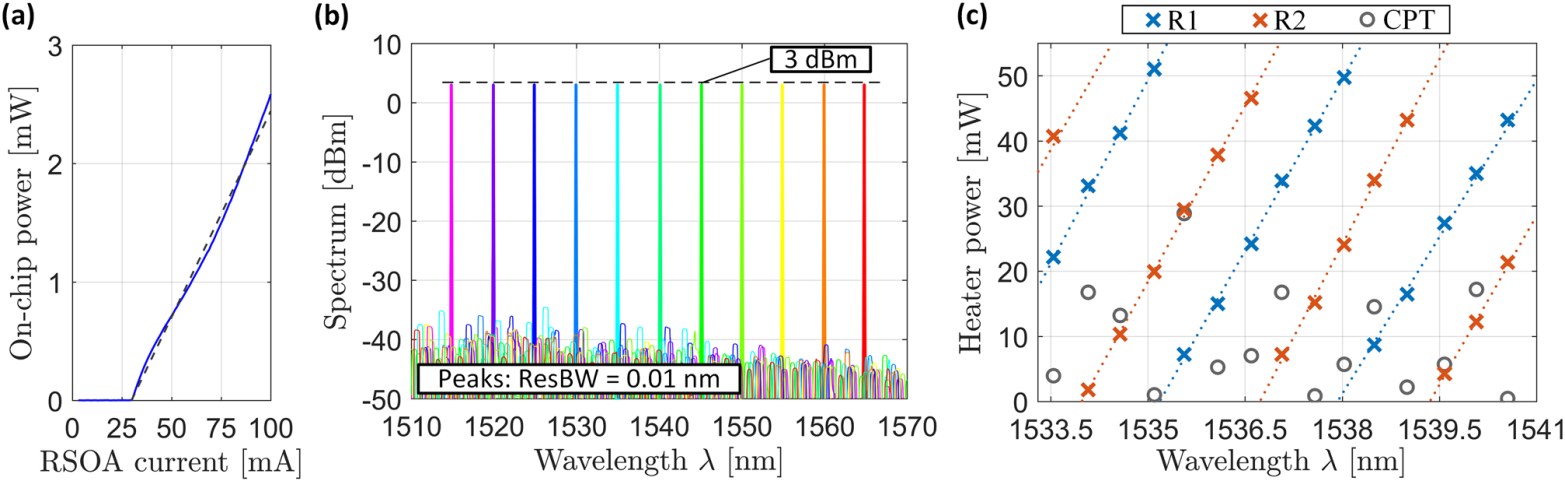
Device characterization results. (**a**) *P*–*I*-curve recorded for lasing operation at 1550 nm. A threshold current of 30 mA and slope efficiency of roughly 35 mW/A are found from the measurement. The output power *P* refers to the on-chip power in the bus waveguide between R2 and GC 4, see Fig. 1b. (**b**) Superimposed lasing spectra recorded in steps of 5 nm within the single-mode tuning range between 1515 and 1565 nm, covering the complete telecommunication C-band. The RSOA bias current was adjusted for each operating point to maintain a constant output power level of 3 dBm. We verify longitudinal single-mode operation by observing the full emission spectrum across the entire RSOA gain bandwidth, finding an SMSR that is consistently better than 40 dB. (**c**) Tuning map of the laser emission, indicating the heater powers of the rings R1 and R2 (blue and red crosses) and of the cavity phase tuner (CPT, grey circles) to reach a certain target wavelength. For better visibility, we only plot a small part of the overall tuning range. The fitted ramps (dotted lines) for the ring heater powers serve as a guide to the eye. The vertical offset of the fitted ramps corresponds to twice the π-power *P*_π_ of the respective ring heater, which is independently measured to be $P_{{\pi ,{\text{R}}1}} = 24.4\;{\text{mW}}$ for R1 and $P_{{\pi ,{\text{R}}2}} = 24.1\;{\text{mW}}$ for R2, see Supplementary Section S4.

For demonstrating the functionality of the device, we select the ECL wavelength by tuning the two ring resonators to a common resonance and by optimizing the cavity phase for maximum output power at GC 1, see Fig. 1b. Once the appropriate tuning parameters are found, they can be stored in a look-up table for later use and for rapid tuning, see Supplementary Section 4. In a first step, we tune the ECL to a wavelength of 1550 nm and measure the *P*–*I*-characteristics, Fig. 5a, where *I* is the pump current and where *P* refers to the on-chip output power in the bus waveguide between R2 and GC 4, see Fig. 1b. The *P*–*I*-curve does not exhibit any kinks that are typical signs of mode hops, even when leaving the cavity phase tuner unchanged while ramping up the current to its final value of 100 mA. Note, however, that such kink-free *P*–*I*-curves require proper initial adjustment of the cavity phase tuner. These findings are in line with the existing literature, where some integrated ECL exhibit mode hops when not adjusting the phase^3,9^, while others do not shown any signs of mode hops over a large range of drive currents^3,5^. We believe that the occurrence of mode hops depends strongly on the specific design of the RSOA, in particular the device length, and on the pump current. Note also that our device is operated only up to a rather modest pump current of 100 mA, limited by imperfect thermal contact of the RSOA and the metal submount. From the measurement in Fig. 5a, we find a threshold pump current of 30 mA and a slope efficiency of 35 mW/A. Note that our current device features four outputs (GC 1–4), Fig. 1b. Using an appropriate design, the emission can be concentrated to a single dominant port, thereby increasing the output power as well as the slope efficiency.

In a second step, we then record lasing spectra within the available tuning range, which covers a bandwidth of $\Delta \lambda_{{{\text{tun}}}} = 50\;{\text{nm}}$ between 1515 and 1565 nm and thus comprises the complete optical telecommunication C-band, see Fig. 5b for a superposition of all recorded spectra. For simplicity, we choose wavelength steps of 5 nm, and we verify longitudinal single-mode operation for each wavelength by recording the full spectrum across the RSOA gain bandwidth. From these spectra, we also extract the SMSR, which exceed 40 dB. The RSOA pump current was chosen to be approximately 100 mA and was then fine-tuned for each operating point to maintain an equal on-chip power level of around 3 dBm in the waveguide leading to GC 1. Figure 5c shows a tuning map of the laser emission, indicating the heater powers for the rings R1 and R2 (blue and red crosses) and for the cavity phase tuner (CPT, grey circles) to reach a certain target wavelength. The fitted ramps (dotted lines) for the ring heater powers serve as a guide to the eye. The vertical offset of the fitted ramps corresponds to twice the π-power *P*_π_ of the respective ring heater, which is independently measured to be $P_{{\pi ,{\text{R}}1}} = 24.4\;{\text{mW}}$ for R1 and $P_{{\pi ,{\text{R2}}}} = 24.1\;{\text{mW}}$ for R2, see Supplementary Section S4. Exploiting the fact that all phase tuners have the same cross section and hence the same tuning efficiency, we estimate a single-pass π-power of approximately $P_{{\pi ,{\text{CPT}}}} = 24\;{\text{mW}}$ for the cavity phase tuner. Note that the heater power for the cavity phase tuner (grey circles) in Fig. 5c does not follow a systematic trend but appears rather random. This is due to the fact that the different emission wavelengths measured for recording the tuning map correspond to different longitudinal modes of the laser cavity. Note also that, for tuning one of the cavity modes to the targeted emission wavelength, it is sufficient to operate the cavity phase tuner in a single-pass phase shift range between 0 and π.

All experiments were performed at room temperature, with the metal submount of the multi-chip assembly placed on a vacuum chuck. Since this vacuum chuck is rather massive, it maintains a constant temperature during operation of the device, even without additional temperature control. Note that photonic wire bonds have been demonstrated to be resilient with respect to temperature changes^23^. Assuming that the temperature-dependence of the RSOA gain and of the operating points of the ring resonators can be accounted for by closed-loop wavelength- and power-control algorithms in combination with on-chip wavelength monitors^28,29^, we believe that the hybrid ECL may eventually be operated without active temperature control of the package. In our experiments, the thermal coupling between the RSOA and the submount leaves room for further improvement. With proper thermal coupling, the device can be operated with a maximum current of 200 mA rather than the 100 mA used in our experiment, which leaves room for further increasing the output power.

**Figure 6:**
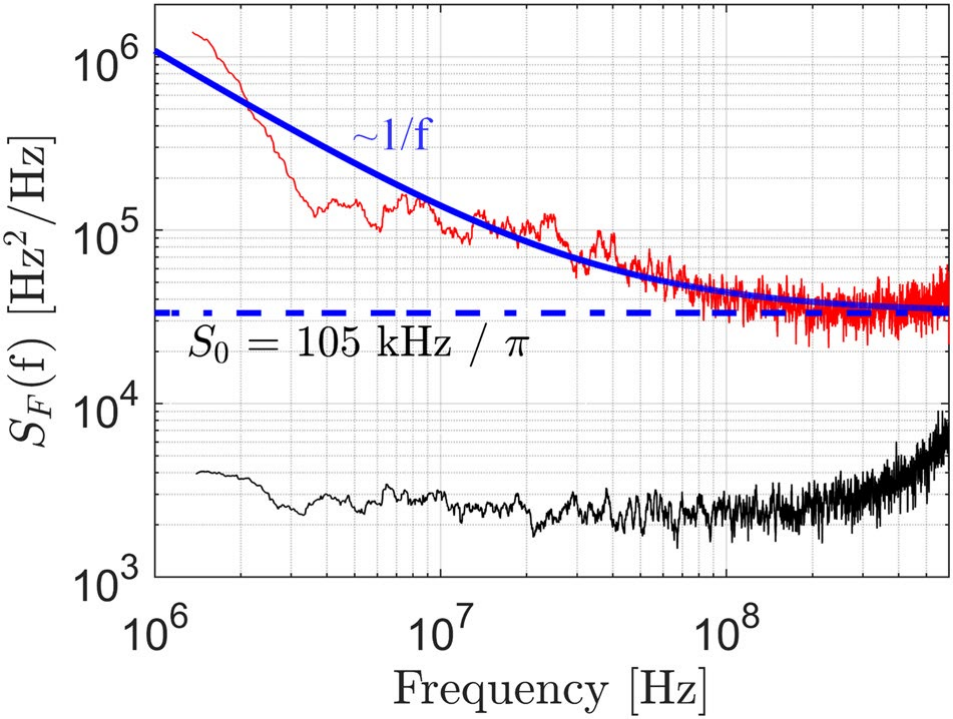
FM-noise spectrum and linewidth measurement. The FM-noise spectrum of the ECL is obtained through heterodyne detection with a narrow-band reference LO laser (Keysight N7714A) and subsequent digital signal processing (red trace). The sampled time-domain waveform comprises 9 × 10^5^ points, recorded at a sampling rate of 256 GSa/s. The spectrum is first smoothed by taking a moving average over 50 neighboring points, and then fitted by a model function of the form $S_{{\text{F}}} (f) = S_{0} + S_{1} f^{ - 1}$ (solid blue line). An instantaneous linewidth of $\delta f = \pi S_{0} \approx 105\;{\text{kHz}}$ is extracted from the spectrally white part of the FM noise spectrum (dashed blue line). In an independent experiment, we also superimpose the tones of two nominally identical LO lasers and extract the FM noise spectrum for each of these lasers (black trace). This measurement confirms that the FM noise level of the LO laser line is at least one order of magnitude below that of the ECL-LO beat note, thereby supporting the assumption that the extracted Lorentzian linewidth of 105 kHz can indeed be attributed to the phase noise of the ECL for the frequency range considered here.

We further measure the phase-noise characteristics of the emitted laser line. To this end, we use a setup similar to the one described in Ref.^30^, which relies on a heterodyne measurement technique^31^. In this approach, the output of the ECL is superimposed with a narrow-linewidth local oscillator (LO) tone in a 90° optical hybrid, and the superimposed signals are then detected by a set of balanced photodetectors and digitized with an oscilloscope. The measurements are carried out at a tuned ECL wavelength close to 1550 nm, where the exact operating point has been fine-tuned for minimum linewidth. The result of the FM-noise spectrum calculation^31^ is shown as a red trace in Fig. 6. The intrinsic (Lorentzian) linewidth is obtained by first fitting a model function of the form $S_{{\text{F}}} (f) = S_{0} + S_{1} f^{ - 1}$ to the measured FM-noise spectrum. This leads to $S_{{0}} = 3.3 \times 10^{4} {\text{ Hz}}$ and to a Lorentzian linewidth of $\delta f = \pi S_{0} \approx 105\;{\text{kHz}}$, indicated by a dashed blue line in Fig. 6. We also measure the phase-noise characteristics of the LO laser (Keysight N7714A) in an independent experiment, where we superimpose the tones of two nominally identical LO lasers and extract the FM noise spectrum of each of them, see black trace in Fig. 6. The FM noise level of the LO laser lines is at least an order of magnitude below that of the ECL-LO beat note, thereby confirming that the extracted Lorentzian linewidth of $\delta f \approx 105\;{\text{kHz}}$ can indeed be attributed to the phase noise of the ECL. To benchmark our measurement, we have theoretically estimated the linewidth that could be expected based on the characteristics of the RSOA and the external feedback circuit, see Supplementary Section 6. The theoretical prediction and the actually measured intrinsic line width are in reasonable agreement.

As another figure of merit, we tried to measure the relative intensity noise (RIN) spectrum of our ECL. The sensitivity of this measurement, however, was limited by the fact that the fiber-coupled output power of the device is rather low. Specifically, as indicated in Fig. 1a, our chip contains unnecessary 2 × 2 MMI before the grating coupler outputs, which themselves feature coupling losses of at least 5 dB. In addition, we used a fiber-coupled circulator with an additional loss of 1.5 dB to avoid back-reflection of light from our measurement setup into the laser. These additional losses limit the sensitivity of the RIN measurement to approximately − 140 dBc/Hz, dictated by the noise floor of our electrical spectrum analyzer (Agilent N9030A). In our measured RIN spectrum, we do not find any peaks in the RIN spectrum that exceed this limit and that could be an indication of reflections from within or outside the laser cavity. Our findings are consistent with published results on hybrid ECL^9,11,32^, in which RIN levels below − 140 dBc/Hz have been regularly obtained.

Note that the coupling losses between the InP gain chip and the SiP chip^22^ as well as the losses in the external-cavity circuit may be further reduced, thereby leaving room for improving the emission power and the linewidth of the ECL. Still, the performance of our current devices is already on par with that of previously demonstrated hybrid ECL that combine standard SiP feedback circuits with InP gain elements through active alignment^5,7,8^. Specifically, these devices exhibit linewidths between 37 kHz and 27 MHz along with tuning ranges between 35 and 95 nm and output powers between 0 and 13 dBm. Generally, the performance of ECL can be improved by optimizing the external feedback circuit. When it comes to linewidth, decreasing the propagation losses and thus increasing the cavity Q-factors is key. On the silicon photonic platform, this can be achieved by using, e.g., low-loss rib waveguides, which are obtained by partial etching of rather thick silicon-on-insulator (SOI) device layers and which allow to reduce the propagation losses down to 0.2 dB/cm. Since optical guidance in rib waveguides is weaker than in the strip waveguides used in our device, the rings usually become larger and the FSR reduces accordingly, which may be compensated by a third ring to maintain wide-band tunability^3^. Using low-loss SiP rib waveguides in combination with three-ring external cavity circuits, heterogeneously integrated ECL with linewidth down to 220 Hz have been demonstrated^3^, along with tuning ranges of 110 nm. Even smaller linewidths down to 40 Hz can be achieved by using ultra-low-loss silicon nitride (SiN) waveguides for the feedback circuit^11^. Exploiting the flexibility of the photonic wire bonding approach, the distinct strengths of advanced SiP or SiN external-cavity circuits may be readily leveraged without re-design of on-chip coupling interfaces.

When it comes to maximizing the output power, external feedback circuits based on silicon photonic (SiP) waveguides face the problem of nonlinear loss due to two-photon absorption (TPA) and subsequent free-carrier absorption (FCA), which eventually limits the intra-cavity power and thereby the overall output power. In fact, the power levels found in our device are already in a regime where nonlinear losses such as TPA and TPA-induced FCA in the rings play a role, see Supplementary Section 5 for a more detailed analysis. This problem can be overcome by using SiN-based feedback circuits, which have led to hybrid ECL that exploit two intra-cavity gain elements to offer record-high output powers^9^ of more than 100 mW along with a tuning range of 100 nm and linewidths around 320 Hz. Another approach to achieve high output powers is to boost the laser emission by an external SOA^6^. In this concept, the photonic wire bonding technique may again offer the advantage of efficient heat sinking, which is particularly crucial for high-power booster SOA.

### Summary

We demonstrated a novel approach to hybrid integrated ECL that exploits 3D-printed photonic wire bonds (PWB) to efficiently connect an InP gain chip to a silicon photonic (SiP) external feedback circuit. Our concept avoids high-precision active alignment of the dies with respect to each other and allows to flexibly leverage the distinct strengths of advanced SiP or SiN-based external-cavity circuits. In a proof-of concept, we demonstrated a first-generation hybrid ECL with a tuning range of more than 50 nm, a side mode suppression ratio (SMSR) above 40 dB, and an intrinsic linewidth of 105 kHz. To the best of our knowledge, our work represents the first demonstration of a chip-scale laser that relies on a 3D-printed coupling element within the cavity. The process of photonic wire bonding can be efficiently automated, thereby paving a path towards efficient mass production of ECL.

## Methods

### RSOA mode-field size and location

Prior to module assembly, the mode-field size of the RSOA as well as the location of the emission spot with respect to a reference point on the RSOA chip were measured to ensure correct dimensioning and placement of the PWB on the chip facet. To this end, we operate the RSOA at large injection currents and investigate its amplified spontaneous emission (ASE) without external resonator. We then assume that the ASE in the high-current limit reliably indicates the mode field and the emission spot that the RSOA will exhibit under lasing conditions in the cavity. In our experiments, the intensity distribution of the mode field at the device facet is captured by an infra-red microscope and processed further to extract size and position of the emitted mode field.

### Fabrication

Fabrication is done in-situ by a two-photon lithography step in a negative-tone photoresist (Vanguard Automation GmbH). We used a self-built lithography system, equipped with a 63 × microscope objective lens (numerical aperture 1.4) and galvanometer mirrors for rapid lateral beam movement. A fs-laser (C-Fiber 780 HP, Menlo) with a pulse length of 58 fs and a repetition rate of 100 MHz serves as lithography light source. Details of the fully automated fabrication are also found in previous publications^23^. The start and the end point and the corresponding local directions of the PWB trajectory are found by scanning and imaging the object with the focused writing beam at low intensity. Upon exposure, the fabricated structure is developed in propylene-glycol-methyl-ether-acetate (PGMEA), flushed with isopropanol, and subsequently blow-dried.

## Supplementary Information

Supplementary Information.
