# Supplementary material for: Hybrid external-cavity lasers (ECL) using photonic wire bonds as coupling elements
Source: Sci Rep. 2021 Aug 12;11:16426. doi: 10.1038/s41598-021-95981-w (PMC8361180; doi:10.1038/s41598-021-95981-w)
Supplement: Supplementary file 1 — Supplementary Information. [file 41598_2021_95981_MOESM1_ESM.pdf]

Hybrid external-cavity lasers (ECL) using photonic wire bonds as coupling elements

# - Supplementary Information -

Yilin Xu[1,2,*], Pascal Maier[1,2,*], Matthias Blaicher[1,2], Philipp-Immanuel Dietrich[1,2,3], Pablo Marin-Palomo[1], Wladislaw Hartmann[1], Yiyang Bao[1], Huanfa Peng[1], Muhammad Rodlin Billah[1,2,3], Stefan Singer[1], Ute Troppenz[4], Martin Moehrle[4], Sebastian Randel[1], Wolfgang Freude[1], Christian Koos[1,2,3]

[1]Institute of Photonics and Quantum Electronics (IPQ), Karlsruhe Institute of Technology (KIT), Engesserstrasse 5, 76131 Karlsruhe, Germany.
[2]Institute of Microstructure Technology (IMT), KIT, Hermann-von-Helmholtz-Platz 1, 76344 Eggenstein-Leopoldshafen, Germany.
[3]Vanguard Automation GmbH, Gablonzer Strasse 10, 76185 Karlsruhe, Germany
[4]Fraunhofer Institute for Telecommunications, Heinrich Hertz Institute (HHI), Einsteinufer 37, 10587 Berlin, Germany
**) both authors contributed equally to this work*

1. Experimental methods for RSOA characterization and bond loss estimation

To measure the small-signal gain and the saturation output power of the RSOA, we launch light to the device and measure the amplified signal using an AR-coated lensed single-mode fiber (SMF), see Inset of Fig. 2(a) of the main manuscript. To determine the coupling loss between the on-chip RSOA waveguide and the lensed SMF, we perform a two-step reference measurement: First, we operate the RSOA without the fiber coupled to it and use an integrating sphere (IS) to measure the overall emitted ASE power as a function of injection current, see Fig. S1(a). Next, the RSOA is coupled to the lensed SMF, and the position of the lensed SMF is left untouched for the remainder of the measurements. The fiber-coupled ASE power is measured with the same integrating sphere, Fig. S1(b). From this measurement, we extract the current-dependent ratio of the power $P_{\mathrm{ASE,SMF}}$ captured by the lensed SMF and the overall ASE power $P_{\mathrm{ASE,tot}}$ emitted by the RSOA and measured by the integrating sphere, see Fig. S1(c). At low currents, this ratio is extremely low, since spontaneous emission occurs into many transverse modes, which are all captured by the IS, whereas only the fundamental quasi-TE and quasi-TM mode is captured by the lensed SMF. With increasing current, the fundamental quasi-TE mode of the RSOA waveguide will experience stronger amplification and thus increasingly dominate the overall emitted ASE power. The ratio $P_{\mathrm{ASE,SMF}}/P_{\mathrm{ASE,tot}}$ thus converges asymptotically to the power-transmission factor $\eta_{\mathrm{RSOA,SMF}}$ for coupling of light between the fundamental quasi-TE mode of the RSOA and the horizontally polarized fundamental mode of the lensed SMF. In our experiment, this fiber-chip coupling loss amounts to $-10\log_{10}\eta_{\mathrm{RSOA,SMF}} = 7.4\ \mathrm{dB}$. Note that this rather high loss is caused by the fact that we intentionally operated the SMF with a working distance larger than the specified one to reduce unwanted spurious back-reflections into the RSOA. Note also that for drive currents of $I = 5$ mA or higher, most of the ASE emitted by the bare RSOA is horizontally polarized and the measured ASE power can hence be attributed to the TE-polarized waveguide modes, see Fig. S1(e).

With the coupling loss at hand, we can now determine the RSOA gain. To this end, we launch a test signal through the SMF, extract the output signal via a circulator (CIRC), and estimate the incoming and the outgoing on-chip power in the quasi-TE mode of the RSOA, see Fig. S1(d) for the corresponding measurement setup. From our measurements, we find a small-signal on-chip gain of 23 dB along with an on-chip saturation output power of 12.5 dBm for a wavelength of $\lambda = 1550\ \mathrm{nm}$ and a drive current of 100 mA, see Fig. 2 of the main manuscript.

To estimate the insertion loss of the photonic wire bond, we again use the fiber-coupled ASE power $P_{\mathrm{ASE,SMF}}$ and compare it to the ASE power $P_{\mathrm{ASE,SiP}}$ in the on-chip silicon photonic waveguide that is directly connected to the photonic wire bond. To extract the ASE power $P_{\mathrm{ASE,SiP}}$ in the on-chip waveguide, we operate the assembled module, see Fig. 1(b) in the main manuscript, with the rings R1 and R2 detuned to one another to avoid feedback into the RSOA. We then measure the ASE spectra through GC 4 and calculate the corresponding power levels $P_{\mathrm{ASE,SiP}}$ in the SiP waveguide directly connected to the PWB by taking into account the wavelength-dependent loss of GC 4 and of the MMI as well as the 0.5 dB of wavelength-independent on-chip propagation loss in the 2.5 mm-long waveguide between the PWB and GC 4. For this analysis, we only consider the ASE power emitted into an approximately 0.8 nm-wide band centered at 1550 nm, which does not contain any resonance of R2. The position of the 0.8 nm-wide band is illustrated by a dashed line in Fig. S1(f). With the power levels $P_{\mathrm{ASE,SiP}}$ and $P_{\mathrm{ASE,SMF}}$ as well as the power transmission $\eta_{\mathrm{RSOA,SMF}}$ between the SMF and the fundamental TE mode of the RSOA at hand, we can then extract the power transmission factor $\eta_{\mathrm{RSOA,SiP}}$ between the fundamental quasi-TE mode of the RSOA and the fundamental quasi-TE mode of the SiP waveguide,

$$\eta_{\mathrm{RSOA,SiP}} = \frac{P_{\mathrm{ASE,SiP}}}{P_{\mathrm{ASE,SMF}}}\eta_{\mathrm{RSOA,SMF}}\,. \tag{S1}$$

We repeated this procedure for various pump currents, see Fig. S1(g), finding an insertion loss of the PWB of $-10\log_{10}\eta_{\mathrm{RSOA,SiP}} \approx (2.1 \pm 0.2)\ \mathrm{dB}$. Note that a small part of the ASE power $P_{\mathrm{ASE,SMF}}$ measured in the fiber should even originate from quasi-TM modes of the RSOA, which cannot be extracted through the highly polarization-sensitive grating coupler (GC 4). To take this effect into account, the measured value of $P_{\mathrm{ASE,SMF}}$ would have to be reduced to only represent the portion coming from quasi-TE modes of the RSOA. This would increase the coupling efficiency according to Eq. (S1) and hence reduce the coupling loss, such that the number specified above may be considered a conservative estimate of the PWB loss. Note

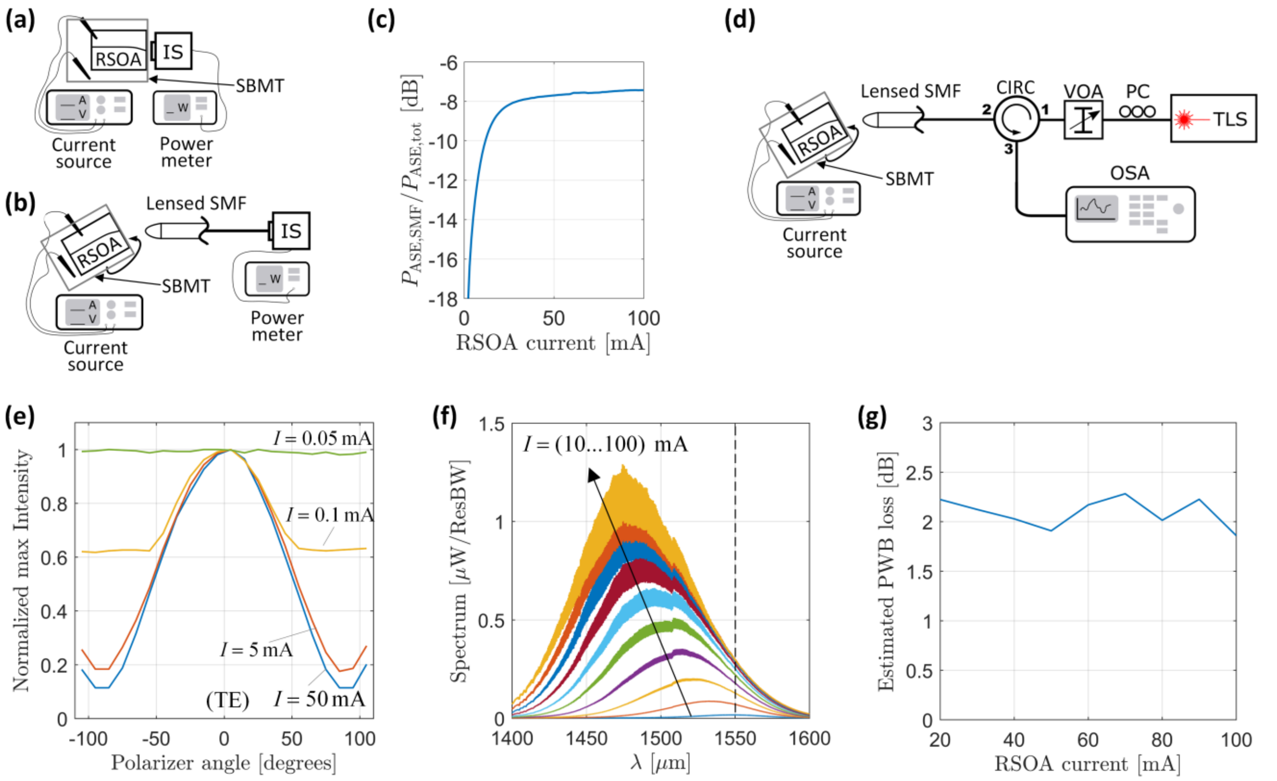

Fig. S1. Experimental setups for RSOA characterization. Abbreviations: SBMT – Submount; CIRC – Optical circulator; IS – Integrating sphere, SMF – Single-mode fiber, TLS – Tunable laser source (Ando AQ4321D), VOA – Variable optical attenuator, OSA – Optical spectrum analyzer (Ando AQ6317B). **(a)** Reference measurement of the overall ASE power $P_{\mathrm{ASE,tot}}$ as function of the drive current. The ASE is captured by an integrating sphere (IS). **(b)** Measurement of the current-dependent ASE power $P_{\mathrm{ASE,SMF}}$ coupled to a lensed single-mode fiber (SMF). **(c)** Current-dependent ratio $P_{\mathrm{ASE,SMF}}/P_{\mathrm{ASE,tot}}$ of the power captured by the lensed SMF and the overall ASE power emitted by the RSOA. In the high-current limit, this ratio converges to the coupling loss between the fundamental quasi-TE mode of the RSOA waveguide and the horizontally polarized fundamental mode of the lensed SMF. **(d)** Measurement setup for small-signal gain, saturation output power and ASE spectrum. A tunable laser source (TLS) and an optical circulator are used to launch test signals to the RSOA and to extract the amplified output signals. **(e)** Polarization-filtered ASE power emitted by the bare RSOA, measured with an infrared microscope and a linear polarizer in the camera path. The abscissa indicates the orientation of the polarizer, where 0° corresponds to maximum transmission for horizontally-polarized light. For drive currents of $I = 5$ mA or higher, most of the emitted ASE is polarized along this direction and may hence be attributed to quasi-TE modes of the on-chip RSOA waveguide. **(f)** ASE power spectra measured through the SMF that is directly coupled to the RSOA, see Subfigure (d). The ASE power levels used for estimating the PWB insertion loss are extracted from a 0.8 nm-wide wavelength band centered at 1550 nm (indicated by a vertical line), i.e., close to the long-wavelength edge of the ASE. In this region, the power spectral density of the ASE saturates for large pump currents, thereby reducing the uncertainty of the measurement technique. **(g)** PWB coupling loss of $-10\log_{10}\eta_{\mathrm{RSOA,SiP}}$ extracted from ASE spectra at different pump currents. From these measurements, we estimate losses of (2.1 ± 0.2) dB .

also that the ASE power is extracted around 1550 nm, close to the long-wavelength edge of the ASE spectrum, see Fig. S1(f). In this region, the power spectral density of the ASE saturates for large pump currents, Fig. S1(f), thereby reducing the uncertainty of the measurement technique and eliminating the impact of spurious lasing lines that might occur at short wavelengths during measurement of the ASE spectrum through the SiP chip.

2. Characterization and modelling of add-drop ring resonators

In Section "Component characterization" of the main manuscript, we describe our ring resonators based on a simple model illustrated in Fig. S2(a)[1,2]. In this representation, the complex amplitudes of the electric mode fields at the input, the drop, the through, and the add ports are denoted as $\underline{E}_{\mathrm{i}}$, $\underline{E}_{\mathrm{d}}$, $\underline{E}_{\mathrm{t}}$, and $\underline{E}_{\mathrm{a}}$, respectively. Due to the bidirectional operation of the ring filter, the coupling zones between the bus waveguides and the ring are designed symmetrically. Assuming for simplicity that the coupling zones are lossless, the device can be described by real-valued amplitude transmission and coupling coefficients τ and κ, where $\kappa^2 + \tau^2 = 1$. The round-trip loss of the waveguide is quantified by a real amplitude transmission factor $a = \exp(-\alpha L/2)$, where α denotes the power loss coefficient of the ring waveguide and where L is the circumference of the ring. In our analysis, we use a positive time dependence, i.e., $\exp(\,\mathrm{j}(\omega t - \beta(\omega) z))$ for a wave propagating at angular frequency ω along the positive *z*-direction with a propagation constant $\beta(\omega)$. We further introduce the round-trip phase $\theta(\omega) = -\beta(\omega) L$. The propagation constant can be approximated by a Taylor series up to the first derivative of β at a center frequency ω_{c}. We define the effective index $n_{\mathrm{e,SiP}} = \beta / k_0$ with the free-space propagation constant $k_0 = \omega / c$, where c denotes the vacuum speed of light. The effective group refractive index is $n_{\mathrm{eg,SiP}} = n_{\mathrm{e,SiP}} + \omega \, \mathrm{d} n_{\mathrm{e,SiP}} / \mathrm{d}\omega$. The complex through-port amplitude transmission $\underline{E}_{\mathrm{t}} / \underline{E}_{\mathrm{i}}$ is then given by[2]

$$\frac{\underline{E}_{\mathrm{t}}(\omega)}{\underline{E}_{\mathrm{i}}(\omega)} = \frac{\tau - a\tau \, \mathrm{e}^{\mathrm{j}\theta(\omega)}}{1 - a\tau^2 \, \mathrm{e}^{\mathrm{j}\theta(\omega)}}, \tag{S2}$$

where

$$\theta(\omega) = -\beta(\omega) L \approx \theta_0 - n_{\mathrm{eg,SiP}} (\omega - \omega_{\mathrm{c}}) \frac{L}{c}, \tag{S3}$$

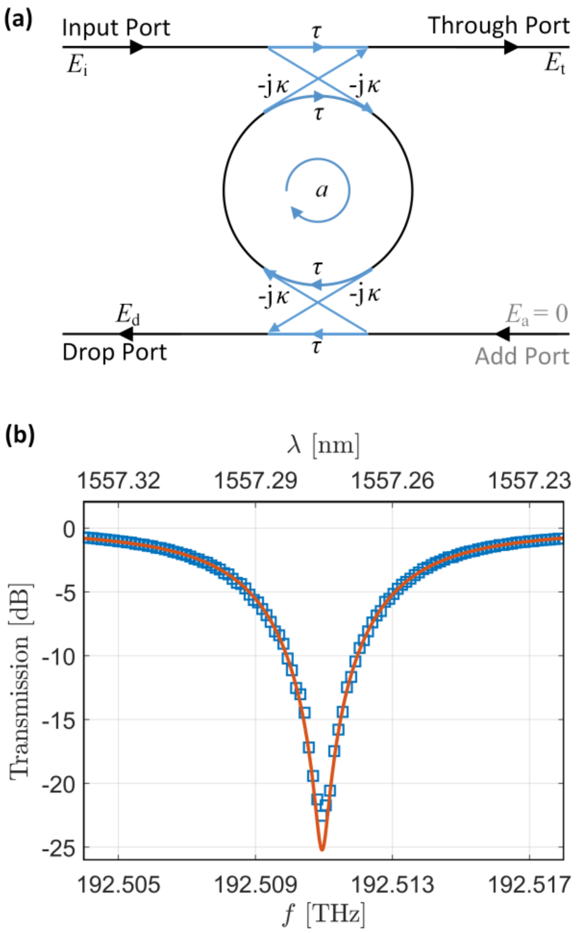

Fig. S2. Schematic of a symmetrically coupled add-drop ring resonator and measured through-port resonance. **(a)** Complex amplitudes of the electrical mode field at input, drop, through and add port are denoted by $\underline{E}_{\mathrm{i}}$, $\underline{E}_{\mathrm{d}}$, $\underline{E}_{\mathrm{t}}$, and $\underline{E}_{\mathrm{a}}$, respectively. The coupling section is described by the amplitude transmission τ and the coupling coefficient κ, which are both assumed to be real-valued with $\kappa^2+\tau^2=1$ for a lossless coupling zone. The round-trip loss of the waveguide is quantified by a real-valued amplitude transmission factor $a=\exp(-\alpha L/2)$, where α denotes the power loss coefficient of the ring waveguide and where L is the circumference of the ring. **(b)** Measured through-port power transmission $\left|\underline{E}_{\mathrm{t}}(\omega)/\underline{E}_{\mathrm{i}}(\omega)\right|^2$ (blue squares) and fit according to Eq. (S2) (orange line), shown for one exemplary ring resonance of R2.

$$\theta_0=-n_{\mathrm{e,SiP}}\,L\frac{\omega_{\mathrm{c}}}{c}. \tag{S4}$$

Note that by suitable choice of reference planes, we opted for real-valued quantities τ and κ, contrary to Ref [2]. Similarly, by considering outcoupling after half a perimeter, the complex drop port amplitude transmission $\underline{E}_{\mathrm{d}}/\underline{E}_{\mathrm{i}}$ is given by[2]

$$\frac{\underline{E}_{\mathrm{d}}(\omega)}{\underline{E}_{\mathrm{i}}(\omega)}=\frac{-\sqrt{a}\kappa^2\,\mathrm{e}^{\mathrm{j}\theta(\omega)/2}}{1-a\tau^2\,\mathrm{e}^{\mathrm{j}\theta(\omega)}}. \tag{S5}$$

From this relation, we can derive the Q-factor of the coupled resonator (loaded Q factor), see Eq. (22) in Ref [3],

$$Q\approx\frac{n_{\mathrm{eg,SiP}}\,\omega L}{2c}\frac{\sqrt{a}\tau}{1-a\tau^2} \tag{S6}$$

We determine the parameters a, τ, $n_{\mathrm{eg,SiP}}$, and θ_0 from through-port power transmission measurements via grating couplers GC 1 and GC 3, see Section "Component characterization" and Fig. 1(b) in the main manuscript. The power transmission measurement is corrected to eliminate the grating-coupler insertion loss, that is independently measured using a dedicated test structure, and Eq. (S2) is then fitted to the extracted power spectrum $\left|\underline{E}_{\mathrm{t}}(\omega)/\underline{E}_{\mathrm{i}}(\omega)\right|^2$. To this end, we directly extract $n_{\mathrm{eg,SiP}}$ from $\mathrm{FSR}=c/(Ln_{\mathrm{eg,SiP}})$ in a first step and use this value to fit Eq. (S2) to the shape of the individual resonances, assuming a constant $n_{\mathrm{eg,SiP}}$. Since the measured data contains resonances of both rings, we first cut out relevant data segments around each resonance and assign them to the corresponding ring. Measurement data and fit curve are shown for one exemplary resonance dip in Fig. S2(b). The phase offset θ_0 in Eq. (3) can be adjusted by the thermal phase tuners and is responsible for the absolute frequency position of the transmission spectra $\left|\underline{E}_{\mathrm{t}}(\omega)/\underline{E}_{\mathrm{i}}(\omega)\right|^2$ and $\left|\underline{E}_{\mathrm{d}}(\omega)/\underline{E}_{\mathrm{i}}(\omega)\right|^2$ on the frequency axis. For the plot in Fig. 3(a) of the main manuscript, θ_0 is adjusted such that the calculated transmission resonances coincide at $\lambda_{\mathrm{c}}=1550\,\mathrm{nm}$. Around the wavelength of 1550 nm, we measure individual free spectral ranges and find FSR of 368.2 GHz and 340.7 GHz for R1 and R2, respectively. This corresponds to $n_{\mathrm{eg,SiP}}\approx 4.18$, in good agreement with platform specifications and with simulations and measurements of waveguides with similar dimensions[4]. We further find $a\approx 0.994$, $\tau\approx 0.973$, $\kappa\approx 0.23$, leading to Q-factors of about 28 000 for each of the rings according to Eq. (S6).

3. Vernier tuning range and tuning enhancement factor

In many cases of practical interest, the tuning range of an ECL with a feedback circuit of two cascaded rings is dictated by the frequency spacing between the main reflection peak and the most prominent side peaks that arise from nearly-overlapping ring resonances. To estimate this frequency spacing, we assume that the two rings R1 and R2 are tuned to a common central resonance frequency f_0 and we express all other resonance frequencies as $f_{\mathrm{R1},m}=f_0+m\times\mathrm{FSR}_1$ for ring R1 and as $f_{\mathrm{R2},m}=f_0+m\times\mathrm{FSR}_2$ for ring R2. The difference between the FSR of the two rings is $\Delta\,\mathrm{FSR}=\mathrm{FSR}_1-\mathrm{FSR}_2$ with $\mathrm{FSR}_1>\mathrm{FSR}_2$ without loss of generality, see Eq. (1) in the main manuscript. We denote the frequency distance of the R2 resonance number m and the R1 resonance number $(m-1)$ with d_m and find

$$\begin{aligned}d_m&=m\times\mathrm{FSR}_2-(m-1)\times\mathrm{FSR}_1\\&=\mathrm{FSR}_1-m\times\Delta\,\mathrm{FSR}\end{aligned}, \tag{S7}$$

$$\Delta\,\mathrm{FSR}=\mathrm{FSR}_1-\mathrm{FSR}_2>0\,. \tag{S8}$$

If $d_m=0$ the resonance frequencies would exactly coincide. This is only possible if $m=\mathrm{FSR}_1/\Delta\,\mathrm{FSR}$ is an integer, i.e., if $T=\mathrm{FSR}_1/\Delta\,\mathrm{FSR}$ in Eq. (1) of the main manuscript happens to be an integer. Otherwise, the modulus $|d_m|$ of the line spacing takes the smallest possible value $\min\{|d_m|\}<\Delta\mathrm{FSR}/2$ when m takes the value of the nearest integer to T, $m=[T]$. This can be mathematically proven by using the inequality $T-\frac{1}{2}<m<T+\frac{1}{2}$ in combination with Eq. (S7), which leads to

$$\min\{|d_m|\}=\left|\mathrm{FSR}_1-[T]\times\Delta\,\mathrm{FSR}\right|<\Delta\,\mathrm{FSR}/2\,. \tag{S9}$$

For a finite resonance linewidth δf, an *imperfect* overlap of two lines as in Eq. (S9) is therefore possible if $\Delta\,\mathrm{FSR}/2 < \delta f$. In this case, we can calculate the index *m* of the resonance of R2, that overlaps with the nearest resonance of index $(m-1)$ of R1 and thus leads to the first pair of side peaks,

$$m = \left[T\right]. \tag{S10}$$

The precise frequency of the side mode, that can occur due to the partially overlapping resonances of R1 and R2, depends on the resonance line shapes of the individual rings and lies in the interval limited by $f_0 + m \times \mathrm{FSR}_2$ and $f_0 + (m-1) \times \mathrm{FSR}_1$. The error when approximating the side mode frequency with either interval limit is smaller than $\Delta\,\mathrm{FSR}/2$.

4. Tuning map

The ECL emission wavelength is selected by aligning the two ring resonators for a common resonance and by optimizing the cavity phase for maximum output power. Once the appropriate tuning parameters are found, they can be stored in a look-up table for later use and for rapid tuning. A simple example of such a tuning map is shown in Fig. S3. When heating only one ring, the corresponding resonance is detuned in proportion to the heating power, while the resonance of the other ring stays nearly constant, indicating very low thermal crosstalk. The vertical offset of the traces obtained from neighboring resonances corresponds to twice the π-power P_π of the respective ring heater, which amounts to $P_{\pi,\mathrm{R1}} = 24.4\ \mathrm{mW}$ for R1 and $P_{\pi,\mathrm{R2}} = 24.1\ \mathrm{mW}$ for R2. To operate the laser at, e.g., 1549 nm, one can choose the heating powers $P_{\mathrm{R1}} \approx 34.8\ \mathrm{mW}$ and $P_{\mathrm{R2}} \approx 25.0\ \mathrm{mW}$ based on the tuning map, see dashed lines in Fig. S3. Due to residual thermal crosstalk, the final operation point needs to be fine-tuned in an iterative approach.

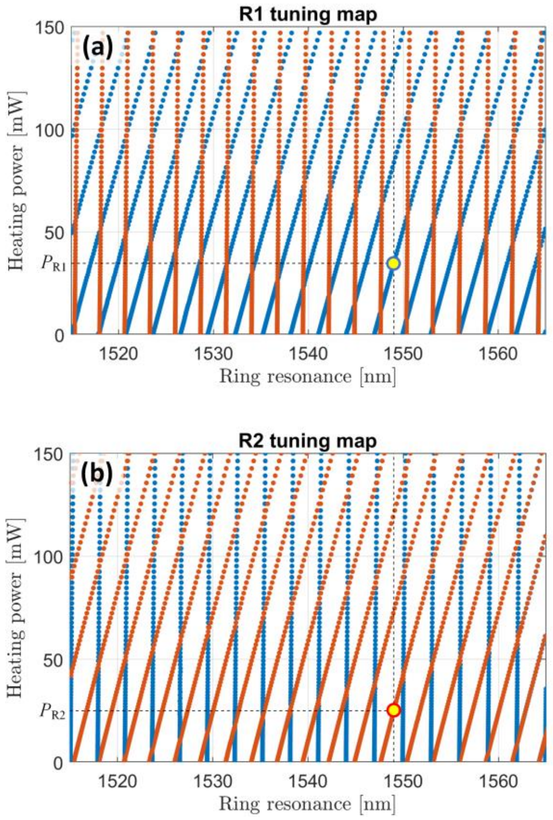

Fig. S3. Tuning map for the ring resonators. The graphs show the wavelengths of the various ring resonances (horizontal axes) for different heating powers (vertical axes) applied to R1 (blue) or R2 (red). The vertical offset of the traces obtained from neighboring resonances corresponds to twice the π-power P_π of the respective ring heater, which is found to be $P_{\pi,\mathrm{R1}} = 24.4\ \mathrm{mW}$ for R1 and $P_{\pi,\mathrm{R1}} = 24.1\ \mathrm{mW}$ for R2. To operate the laser at, e.g., $\lambda_0 = 1549\ \mathrm{nm}$ (vertical dashed black lines), one can choose the heating powers $P_{\mathrm{R1}} \approx 34.8\ \mathrm{mW}$ and $P_{\mathrm{R2}} \approx 25.0\ \mathrm{mW}$ (horizontal dashed black lines). Note that the tuning range of each ring exceeds the corresponding FSR such that each emission wavelength can be reached by different sets of tuning powers. **(a)** Resonance detuning when heating only R1. The resonance of R1 (blue) is detuned in proportion to the heating power, while the resonance of R2 (red) stays nearly constant, indicating low levels of thermal crosstalk. **(b)** Resonance detuning when heating only R2. The resonance of R2 (red) is detuned in proportion to the heating power, while the resonance of R1 (blue) stays almost constant.

5. Two-photon absorption (TPA) and TPA-induced free carrier absorption in the external cavity circuit

The high optical power in the ring-resonator waveguides of the silicon photonic (SiP) external-cavity circuit might lead to detrimental nonlinear effects. To quantify these effects at least approximately, we have performed further estimations and measurements. The power enhancement in a ring resonator as compared to the power in the corresponding bus waveguide can be described by the buildup factor *B*, see Eq. (2.34) in Ref [2],

$$B = \frac{\kappa^2}{\left(1 - a\tau^2\right)^2}. \tag{S11}$$

For our ring resonators, we estimate a round-trip transmission factor of $a = 0.994$ along with transmission and coupling factors $\tau = 0.973$ and $\kappa = 0.23$ for the two coupling zones, see Supplementary Section 2, which leads to a build-up factor of $B = 15.2$, corresponding to 11.8 dB. We further consider the maximum emission power of approximately 15 dBm that the RSOA reaches in the limit of high input powers – slightly more than the saturation output power of 12.5 dBm, that was defined by the 3 dB gain compression point, see Fig. 2(a) in the main manuscript. Taking into account the insertion loss of approximately 2.1 dB of the PWB, see Supplementary Section 1, the on-chip insertion loss of approximately 3 dB of the 2×2 MMI, and a single-pass loss of approximately 0.4 dB of the 2.2 mm long on-chip strip waveguide, we hence estimate an on-chip

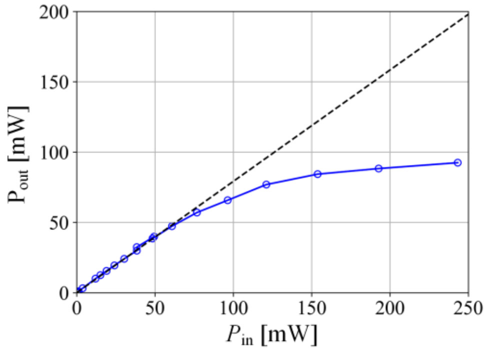

Fig. S4. Reference transmission measurement of a 2.25-mm-long straight silicon photonic waveguide with the same (500 nm × 220 nm) cross-section as used in the ECL external cavity circuit. P_{in} refers to the on-chip power level right after the input grating coupler, P_{out} to the on-chip power before the output grating coupler. The measured trace (blue) starts to deviate from the linear fit at low power levels (black dashed line) at input powers around 80 mW (19 dBm).

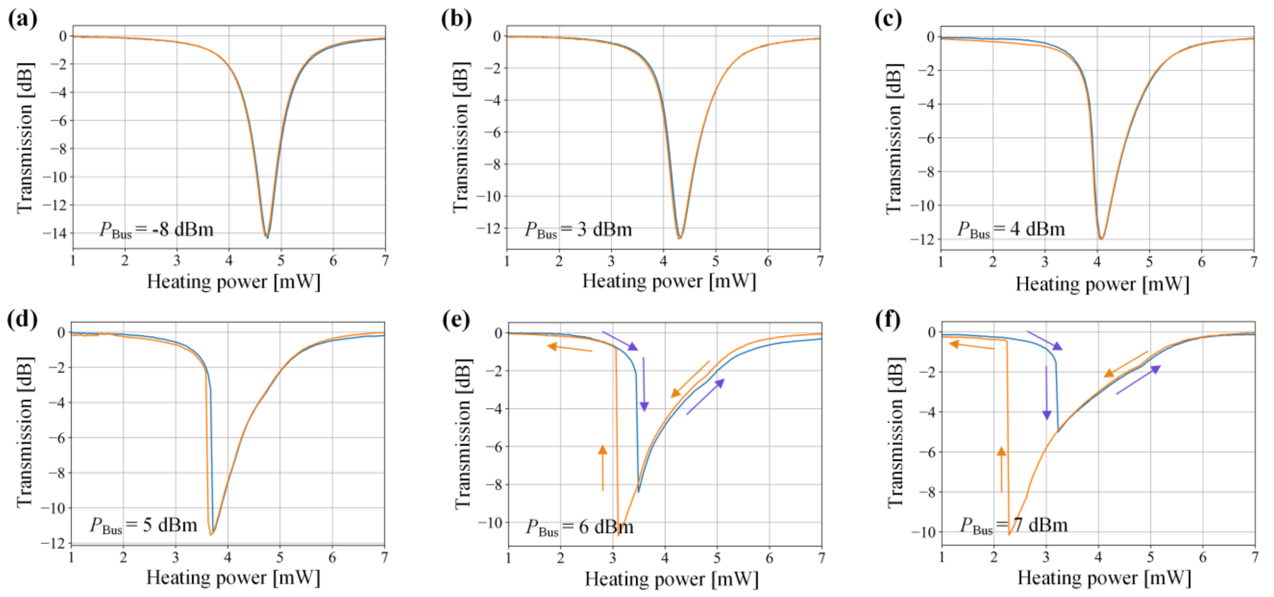

Fig. S5. Transmission measurements through ring R1 between grating couplers GC 1 and GC 3 at different optical input power levels P_{Bus} in the bus waveguide. To avoid destroying the ECL during the experiment, we used a SiP chip that is nominally identical to the one contained in the ECL and that was fed by a test laser and an EDFA. We tune the laser to an emission wavelength slightly below a resonance of ring R1 and then ramp the heater current of ring R1 up and down to scan the resonance across the fixed laser wavelength while measuring the output power. This experiment is repeated at different levels of the on-chip input power P_{Bus}. Starting from a power level P_{Bus} of 4 dBm, we first find that the resonance peaks become increasingly skewed, see Subfigure (c). At around 5 dBm, we further observe the onset of hysteresis and bi-stable behavior, which becomes more pronounced with increasing power, Subfigures (d),(e),(f). At the same time, we find that the depth of the transmission dip decreases, see Fig. R3 for details. Heating of the ring due to linear absorption may explain skewing and bi-stability[5], but the reduction of the depth of the resonance dips is a clear indication for nonlinear losses such as TPA and TPA-induced FCA in the rings[6].

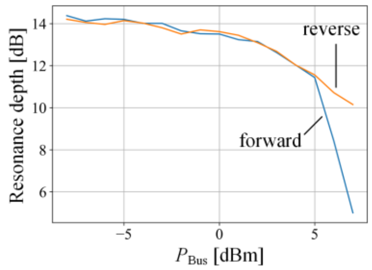

Fig. S6. Resonance depths for different optical power levels P_{Bus} in the bus waveguide, extracted from transmission measurements between grating couplers GC 1 and GC 3, see Fig. S5. The resonance depths start to decrease already at power levels around 0 dBm in the bus waveguide. At around 5 dBm, we observe the onset of hysteresis and bi-stable behavior, which becomes more pronounced with increasing power. The blue trace shows the resonance depths recorded when ramping the heating power up ("forward") and the orange trace shows the ones when ramping the heating power down ("reverse").

power of approximately 9 dBm that is fed to each of the rings through the bus waveguide connected to the MMI. Assuming an unperturbed resonator in perfect resonance, the power propagating into a single direction within the rings would then amount to more than 19.5 dBm. The overall intra-cavity power is even higher, since the rings are fed from both sides. To estimate the impact of nonlinear losses, we have measured the power-dependent transmission characteristics of a 2.25 mm-long straight silicon photonic waveguide with a cross section of 500 nm × 220 nm that is identical to that of our ring resonator. The results are shown in Fig. S4, exhibiting signs of nonlinear losses such as TPA and TPA-induced FCA for on-chip powers of approximately 19 dBm (80 mW). We should hence expect that the rings of our silicon photonic feedback circuit are affected by such effects as well.

We experimentally confirm this notion by conducting transmission measurements between grating couplers GC 1 and GC 3 of our ECL chip at different optical power levels. To avoid destroying the ECL during the experiment, we used a SiP chip that is nominally identical to the one contained in the ECL and that was fed by a test laser and an EDFA. We tune the laser to an emission wavelength slightly below a resonance of ring R1 and then ramp the heater current of ring R1 up and down to scan the resonance across the fixed laser wavelength while measuring the output power. Figure S5 shows the on-chip transmission through the bus waveguide of the ring resonator, measured as a function of heater power for different optical input power levels P_{Bus} . For increasing laser power, we make three observations similar to Fig. 20 of Ref [3]: Starting from a power level P_{Bus} of 4 dBm, we first find that the resonance peaks become increasingly skewed, see Fig. S5(c). At around 5 dBm, we further observe the onset of hysteresis and bi-stable behavior, which becomes more pronounced with increasing power, Fig. S5(d,e,f). At the same time we find that the depth of the transmission dip decreases, Fig. S5 – an effect that sets on already at power levels around 0 dBm in the bus waveguide. Heating of the ring due to linear absorption may explain skewing and bi-stability[5], but the reduction of the depth of the resonance dips is a clear indication of nonlinear losses such as TPA and TPA-induced FCA in the rings[6]. These experimental findings are in reasonable agreement with the estimated build-up factor B of 11.8 dB and the fact that nonlinear losses in silicon nanowire waveguides start to become relevant at power levels around 19 dBm, see Fig. S4. Based on these estimations, we should expect that operation of our device is not only subject to heating of the rings due to linear optical intra-cavity losses, but also to TPA and TPA-induced FCA in the ring – at least to some degree. In our experiments, we did not encounter any detrimental effects such as hysteretic wavelength-

tuning behavior or unwanted pulsation of the laser emission. Still, implementing ECL with higher emission power might either require large-area SiP waveguides[7], active removal of free carriers by reverse-biased p-i-n junctions integrated into the ring waveguide[8,9], or feedback circuits base on waveguides made from large-bandgap silicon dioxide[10] or silicon nitride[11,12].

6. Theoretical discussion of the ECL linewidth

As a reference for the experimentally measured phase-noise properties of our ECL, we theoretically estimate the linewidth that could be expected based on the characteristics of the RSOA and the external feedback circuit. To this end, we follow the formalism described in Ref [7,11,13], which is based on Ref [14]. In this model, the laser is simplified to an active section in between two reflecting facets. The back facet has a frequency-independent amplitude reflection coefficient coefficient r_b, while the front facet is represented by a frequency-dependent complex amplitude reflection coefficient $\underline{r}_\mathrm{eff}(\omega) = |r_\mathrm{eff}(\omega)|\,\mathrm{e}^{\mathrm{j}\varphi(\omega)}$. The entire external feedback circuit is hence lumped into this frequency-dependent complex reflection factor. The magnitude $|r_\mathrm{eff}(\omega)|$ of the amplitude reflection coefficient accounts for the round-trip losses caused by the PWB and the SiP access waveguides, as well as for the amplitude reflection $|r_\mathrm{mirror}(\omega)|$ of the on-chip Sagnac loop mirror, containing the Vernier ring pair. Neglecting any imbalance of the associated 2×2 MMI, the magnitude $|r_\mathrm{eff}(\omega)|$ of the amplitude reflection coefficient can be written as

$$\begin{aligned}|r_\mathrm{eff}(\omega)| &= \sqrt{\eta_\mathrm{PWB}^2}\,\sqrt{\eta_\mathrm{SiP}^2}\,|r_\mathrm{mirror}(\omega)| \\ &= \eta_\mathrm{PWB}\,\eta_\mathrm{SiP}\,|r_\mathrm{mirror}(\omega)|\end{aligned}, \tag{S12}$$

$$|r_\mathrm{mirror}(\omega)| = \left|\frac{\underline{E}_\mathrm{d,R1}(\omega)}{\underline{E}_\mathrm{i,R1}(\omega)}\right|\left|\frac{\underline{E}_\mathrm{d,R2}(\omega)}{\underline{E}_\mathrm{i,R2}(\omega)}\right|. \tag{S13}$$

The quantities $\underline{E}_\mathrm{d,R1}$, $\underline{E}_\mathrm{i,R1}$, $\underline{E}_\mathrm{d,R2}$, $\underline{E}_\mathrm{i,R2}$ refer to the complex amplitudes of the electric fields at the input and the drop ports of the two rings, see Supplementary Section 2 for details. For the PWB, we find $\eta_\mathrm{PWB} = 61.6\%$ (2.1 dB loss), see Supplementary Section 1. The power transmission $\eta_\mathrm{SiP} = 88.7\%$ through the passive SiP waveguides is calculated from a waveguide length of $L_\mathrm{SiP} = 2.6$ mm and a waveguide loss parameter of $\alpha_\mathrm{SiP} = 2$ dB/cm. The drop-port transmissions $\underline{E}_\mathrm{d,R1}(\omega)/\underline{E}_\mathrm{i,R1}(\omega)$ and $\underline{E}_\mathrm{d,R2}(\omega)/\underline{E}_\mathrm{i,R2}(\omega)$ are calculated for the individual rings according to Supplementary Eq. (S5). The phase $\varphi(\omega)$ of the reflection coefficient accounts for the propagation through the on-chip Si transport waveguides, as well as for the frequency-dependent phase shift associated with the transmission through each of the rings,

$$\begin{aligned}\varphi(\omega) &= \varphi_0 - n_\mathrm{eg,SiP}(\omega-\omega_\mathrm{c})\frac{2L_\mathrm{SiP}}{c} \\ &+ \arg\left\{\frac{\underline{E}_\mathrm{d,R1}(\omega)}{\underline{E}_\mathrm{i,R1}(\omega)}\right\} + \arg\left\{\frac{\underline{E}_\mathrm{d,R2}(\omega)}{\underline{E}_\mathrm{i,R2}(\omega)}\right\}\end{aligned}. \tag{S14}$$

For simplicity, we have neglected the phase shift within the photonic wire bond, which is only 200 µm long, and within the 2×2 MMI.

The frequency-dependent phase of the reflection factor $\underline{r}_\mathrm{eff}(\omega)$ of the external feedback circuit reduces the linewidth δf compared to the linewidth δf_0 of a Fabry-Pérot diode laser with mirror amplitude reflection factors of r_b and $|r_\mathrm{eff}(\omega)|$. This reduction can be quantified[14] by a factor $F^2 = \delta f_0 / \delta f$, which accounts for the local frequency dependence of the amplitude and the phase of $\underline{r}_\mathrm{eff}(\omega)$ as well as for the Henry factor α_H and the photon round-trip time $\tau_\mathrm{0,RSOA} = 2n_{eg,\mathrm{RSOA}}L_\mathrm{RSOA}/c$ in the RSOA with the effective group refractive index $n_{eg,\mathrm{RSOA}}$,

$$F = 1 + A + B, \tag{S15}$$

where

$$A = -\frac{1}{\tau_\mathrm{0,RSOA}}\frac{\mathrm{d}\varphi(\omega)}{\mathrm{d}\omega}, \tag{S16}$$

$$B = \frac{\alpha_\mathrm{H}}{\tau_{0,a}}\frac{\mathrm{d}\ln|r_\mathrm{eff}(\omega)|}{\mathrm{d}\omega}. \tag{S17}$$

For simplicity, we assume that the resonance frequencies of both rings are tuned to perfectly coincide at or near the lasing frequency, leading to a reflection spectrum as shown in Fig. 3(b) of the main manuscript. Tuning of the intra-cavity phase shifts the frequency of the lasing resonator mode with respect to the peak of the reflection spectrum of the external feedback circuit, and the exact linewidth depends on the detuning between the laser emission frequency and the peak of the reflection spectrum.

The Fabry-Pérot linewidth δf_0 depends on the total output power P_o, the population inversion factor n_sp, the internal loss α_i of the RSOA (in units of cm^{-1}), which accounts for excess losses of the active waveguide without the contribution from band-to-band transitions, and the distributed loss α_R, that represents the partially reflective mirrors,

$$\begin{aligned}\alpha_\mathrm{R}(\omega) &= -\frac{1}{2L_\mathrm{RSOA}}\ln\left(r_\mathrm{b}^2|r_\mathrm{eff}(\omega)|^2\right) \\ &= -\frac{1}{L_\mathrm{RSOA}}\ln\left(r_\mathrm{b}|r_\mathrm{eff}(\omega)|\right)\end{aligned}. \tag{S18}$$

With these numbers at hand, we can use the estimation of Henry[15] for the Fabry-Pérot linewidth,

$$\begin{aligned}\delta f_0 = \frac{1}{8\pi}&\frac{\left(cn_\mathrm{eg,RSOA}\right)^2 hf\, n_\mathrm{sp}(\alpha_\mathrm{i}+\alpha_\mathrm{R})\alpha_\mathrm{R}}{P_\mathrm{o}} \\ &\times\left(1+\alpha_\mathrm{H}\right)^2\end{aligned}, \tag{S19}$$

which then leads to the estimated ECL linewidth

$$\delta f = \frac{\delta f_0}{F^2}. \tag{S20}$$

Equations (S12)…(S20) allow to estimate the linewidth of our ECL, using numerical values for the various parameters as specified in Table S1. For the Henry factor, we consider a typical value range of $\alpha_\mathrm{H} = 2...7$ for InP/InGaAsP lasers at a wavelength of 1.5 µm, see Ref [16]. For the population inversion

Table S1. Summary of parameters and values used for the theoretical calculation of the ECL linewidth.

Symbol	Description	Value / extracted from
hf	Photon energy	0.80 eV at a center frequency of f = 193.41 THz
η_{PWB}	Power transmission of the photonic wire bond	58.9…64.6% (2.1 dB±0.2 dB), see Supplementary Section 1
L_{SiP}	Length of passive waveguides on the SiP chip	2.6 mm (2.2 mm transport waveguide between the photonic wire bond and the MMI, see “Results and Discussion”, Section “Device Concept” of the main manuscript; and 0.4 mm of bus waveguide within the Sagnac loop)
η_{SiP}	Power transmission of SiP WG	88.7% (2.6 mm-long silicon photonic waveguide with propagation loss of 2 dB/cm, according to foundry specifications)
a	Round-trip amplitude transmission factor of ring R1 and R2	0.994, see Supplementary Section 2
τ	Amplitude transmission coefficient of the coupling zones of ring R1 and R2	0.973, see Supplementary Section 2
κ	Ampltiude coupling coefficient of the coupling zones of ring R1 and R2	0.23, see Supplementary Section 2
$n_{\text{eg,SiP}}$	Effective (modal) group refractive index of the on-chip SiP waveguides	4.18, see Supplementary Section 2
$n_{\text{eg,RSOA}}$	Effective (modal) group refractive index of the active RSOA section	3.422, from gain ripple frequency spacing, see Fig. 2
L_{RSOA}	Length of RSOA	600 µm, see “Results and Discussion”, Section “Device Concept” of the main manuscript
α_{H}	Henry factor of the RSOA	2…7, typical range[16]
P_{o}	Total output power	10 dBm, assuming equal amount of power from GC1-4
n_{sp}	Population inversion factor	1.25…1.75, typical range[17]
α_{i}	Internal loss of the RSOA	14…20 cm^{-1}, estimated range
r_{b}	RSOA back facet amplitude reflection factor	$\sqrt{90\%}$, see “Results and Discussion”, Section “Component Characterization” of the main manuscript

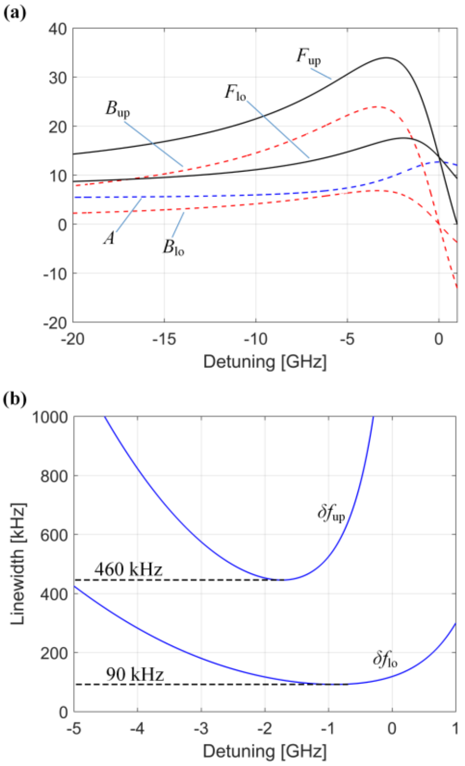

Fig. S7. Theoretically estimated linewidths of the ECL emission. **(a)** Calculated parameters A, B, and F according to Eqs. (S15) – (S17). To account for the uncertainties of the various input parameters listed in Table S1, we plot two traces for B, F, and δf, indicating the corresponding lower (subscript “lo”) and the upper (subscript “up”) boundary. **(b)** Corresponding linewidth according to Eqs. (S19) and (S20). Assuming optimum detuning, we expect linewidths between 90 kHz and 460 kHz. This is in reasonable agreement with the experimentally measured linewidth of approximately 105 kHz.

factor, we consider a typical range of $n_{\text{sp}} = 1.25...1.75$, see p. 198 of Ref [17]. The internal loss of the RSOA is estimated to be in the range of $\alpha_{\text{i}} = (14...20)\ \text{cm}^{-1}$. Figure S7 shows the calculated parameters A, B, and F according to Eqs. (S15) – (S17) and the corresponding linewidth according to Eqs. (S19) and (S20), all as a function of the detuning of the laser emission from the resonance frequency of the two rings. To account for the uncertainties of the various input parameters, we plot two traces for each B, F, and δf, indicating the corresponding lower (subscript “lo”) and the upper (subscript “up”) boundary. Assuming optimum detuning, we expect linewidths between 90 kHz and 460 kHz. This is in reasonable agreement with the experimentally measured linewidth of approximately 105 kHz. Note, however, that the calculation model does not account for any potential impact of nonlinear losses such as two-photon-absorption (TPA) and TPA-induced free-carrier absorption within the rings that were found to potentially play a role in our devices, see Supplementary Section 5.

Acknowledgements

This work was supported by the Deutsche Forschungsgemeinschaft (DFG, German Research Foundation) under Germany’s Excellence Strategy via the Excellence Cluster 3D Matter Made to Order (EXC-2082/1-390761711) as well as through the Collaborative Research Centre (CRC) WavePhenomena (# 1173), by the Bundesministerium für Bildung und Forschung (BMBF) within the joint project PRIMA (# 13N14630) and the project DiFeMiS (# 16ES0948), which is part of the programme “Forschungslabore Mikroelektronik Deutschland (ForLab), by the European Research Council (ERC Consolidator Grant ‘TeraSHAPE’, (# 773248), by the H2020 Photonic Packaging Pilot Line PIXAPP (# 731954), by the Karlsruhe School of Optics and Photonics (KSOP), and by the Alfried Krupp von Bohlen und Halbach Foundation.

Competing interests

P.-I.D. and C.K. are co-founders and shareholders of Vanguard Photonics GmbH and Vanguard Automation GmbH, start-up companies engaged in exploiting 3D nanoprinting in the field of photonic integration and assembly. P.-I.D. and M.R.B. are employees of Vanguard Automation GmbH. Y.X., P.M., M.B., P.-I.D., M.R.B., and C.K. are co-inventors of patents owned by Karlsruhe Institute of Technology (KIT) in the technical field of the publication. M.B. is now an employee of Nanoscribe GmbH, a company selling 3D lithography systems.

References

1. Yariv, A. Universal relations for coupling of optical power between microresonators and dielectric waveguides. *Electron. Lett.* **36**, 321–322 (2000).
2. Rabus, D. G. Ring Resonators: Theory and Modeling. in *Integrated Ring Resonators* 3–40 (Springer, 2007). doi:10.1007/978-3-540-68788-7_2
3. Bogaerts, W. *et al.* Silicon microring resonators. *Laser Photon. Rev.* **6**, 47–73 (2012).
4. Dulkeith, E., Xia, F., Schares, L., Green, W. M. J. & Vlasov, Y. A. Group index and group velocity dispersion in silicon-on-insulator photonic wires. *Opt. Express* **14**, 3853–3863 (2006).
5. Carmon, T., Yang, L. & Vahala, K. J. Dynamical thermal behavior and thermal self-stability of microcavities. *Opt. Express* **12**, 4742–4750 (2004).
6. Xiang, C. *et al.* Effects of nonlinear loss in high-Q Si ring resonators for narrow-linewidth III-V/Si heterogeneously integrated tunable lasers. *Opt. Express* **28**, 19926–19936 (2020).
7. Tran, M. A. *et al.* Ring-resonator based widely-tunable narrow-linewidth Si/InP integrated lasers. *IEEE J. Sel. Top. Quantum Electron.* **26**, 1–14 (2020).
8. Turner-Foster, A. C. *et al.* Ultrashort free-carrier lifetime in low-loss silicon nanowaveguides. *Opt. Express* **18**, 3582–3591 (2010).
9. Trocha, P. *et al.* Analysis of Kerr comb generation in silicon microresonators under the influence of two-photon absorption and fast free-carrier dynamics. *Phys. Rev. A* **103**, 63515 (2021).
10. Takeuchi, T., Takahashi, M., Suzuki, K., Watanabe, S. & Yamazaki, H. Wavelength tunable laser with silica-waveguide ring resonators. *IEICE Trans. Electron.* **E92-C**, 198–204 (2009).
11. Boller, K.-J. *et al.* Hybrid integrated semiconductor lasers with silicon nitride feedback circuits. *Photonics* **7**, 4 (2019).
12. Fan, Y. *et al.* Hybrid integrated InP-Si_3N_4 diode laser with a 40-Hz intrinsic linewidth. *Opt. Express* **28**, 21713–21728 (2020).
13. Kita, T., Tang, R. & Yamada, H. Narrow Spectral Linewidth Silicon Photonic Wavelength Tunable Laser Diode for Digital Coherent Communication System. *IEEE J. Sel. Top. Quantum Electron.* **22**, 23–34 (2016).
14. Kazarinov, R. & Henry, C. The relation of line narrowing and chirp reduction resulting from the coupling of a semiconductor laser to passive resonator. *IEEE J. Quantum Electron.* **23**, 1401–1409 (1987).
15. Henry, C. Theory of the linewidth of semiconductor lasers. *IEEE J. Quantum Electron.* **18**, 259–264 (1982).
16. Osinski, M. & Buus, J. Linewidth broadening factor in semiconductor lasers – An overview. *IEEE J. Quantum Electron.* **23**, 9–29 (1987).
17. Coldren, L. A., Corzine, S. W. & Mashanovitch, M. L. *Diode Lasers and Photonic Integrated Circuits*. (Wiley, 2012).
